# Synthesis and Enzymatic Degradation of Sustainable Levoglucosenone-Derived Copolyesters with Renewable Citronellol Side Chains

**DOI:** 10.3390/polym14102082

**Published:** 2022-05-20

**Authors:** Sami Fadlallah, Quentin Carboué, Louis M. M. Mouterde, Aihemaiti Kayishaer, Yasmine Werghi, Aurélien A. M. Peru, Michel Lopez, Florent Allais

**Affiliations:** URD Agro-Biotechnologies Industrielles (ABI), CEBB, AgroParisTech, 3 Rue des Rouges-Terres, 51110 Pomacle, France; louis.mouterde@agroparistech.fr (L.M.M.M.); aihemaiti.kayishaer@gmail.com (A.K.); yasmine.werghi1@gmail.com (Y.W.); aurelien.peru@agroparistech.fr (A.A.M.P.); michel.lopez@agroparistech.fr (M.L.)

**Keywords:** sustainability, levoglucosenone, oxa-Michael addition, cross-linkable polymers, renewable polyesters, biodegradation

## Abstract

Recently, a renewable five-membered lactone containing citronellol (HBO-citro) was synthesized from levoglucosenone (LGO). A one-pot two-step pathway was then developed to produce a mixture of 5- and 6-membered Lactol-citro molecules (5ML and 6ML, respectively) from HBO-citro. Proton nuclear magnetic resonance (^1^H NMR) of a mixture of 5ML and 6ML at varying temperatures showed that the chemical shifts of the hydroxyls, as well as the 5ML:6ML ratio, are temperature-dependent. Indeed, a high temperature, such as 65 °C, led to an up-field shielding of the hydroxyl protons as well as a drop in the 5ML:6ML ratio. The monomers 5ML and 6ML were then engaged in polycondensation reactions involving diacyl chlorides. Renewable copolyesters with low glass transition temperatures (as low as −67 °C) and cross-linked citronellol chains were prepared. The polymers were then hydrolyzed using a commercial lipase from *Thermomyces lanuginosus* (Lipopan^®^ 50 BG). A higher degradation rate was found for the polymers prepared using Lactol-citro molecules, compared to those obtained by the polycondensation reactions of diacyl chlorides with Triol-citro—a monomer recently obtained by the selective reduction of HBO-citro.

## 1. Introduction

While converting biomass waste into high-performance renewable materials reduces fossil fuel use and greenhouse gas emissions [1,2], it does not solve the environmental problems caused by the accumulation of bioplastic waste [3]. Indeed, the prefix “bio” is likely to be misunderstood by producers/consumers. Bio-based polymers are products that are partially or fully derived from biomass. However, bio-based polymers can be either biodegradable or not [4,5]. Biodegradation is the decomposition of a specific material into final metabolic compounds, such as CO_2_, through an in vivo or in vitro enzyme-initiated/mediated mechanism [6]. Thus, not all bio-based polymers are biodegradable. For these reasons, it is of utmost importance to consider the biodegradation of polymers obtained from biomass as a primary criterion in the evaluation of the sustainability of the corresponding materials. Pellis et al. have recently highlighted the synergy between chemistry and biotechnology to produce new generations of bio-based polymers with environmental and social benefits [7]. Indeed, the biodegradation of synthesized renewable polymers is barely described in the literature. From a general point of view, the (bio)degradation of a macromolecule/polymer is mainly affected by the following characteristics: optical purity [8], crystallinity [9], molecular weight [10] or chemical structure [11].

Polyesters constitute a highly versatile class of polymers that meet the market requirements in terms of production cost and desirable qualities [12]. For example, polyesters are widely used for making textiles, such as dress materials [13]. On the other hand, polyesters are polymers of repeating units containing ester groups that can undergo hydrolysis under certain circumstances. This facilitates their enzymatic degradation if they are susceptible to it [14]. The presence of aliphatic moieties in polyesters allows their biodegradability. Aromatic-containing polymers, such as bio-polyethylene terephthalate (PET) [15], the leading bioplastic in the market, suffer from their resistance to degradation when accumulated in the environment. In general, biodegradable aromatic/aliphatic polyesters typically contain a large fraction of aliphatic ester groups. Above a threshold, which depends on the properties of each polymer, the presence of aromatic groups decreases, or even suppresses, the biodegradability [16].

On the other hand, one of the key challenges in the field of sustainable polymers is to produce renewable materials that can compete with their fossil fuel-based counterparts [17,18,19]. A discussion was recently held to identify the most important topics that should be addressed over the next 100 years in polymer science [20]. Three major topics were identified: (i) new properties and applications, (ii) new synthetic methods, and (iii) sustainability. In this context, functional polymers offer great potential to be used in widespread applications [21,22]. Allcock et al. took advantage of citronellol, a natural acyclic monoterpenoid found in citronella oil [23], to synthesize polyphosphazenes containing citronellol side groups, as potential candidates for ligament and tendon tissue engineering [24]. Hydrolysis experiments in deionized water at 37 °C showed a mass loss of 8–16% and a decrease in molecular weight in the range of 28–88% over 12 weeks. The same authors prepared polyphosphazenes with pendant amino acid citronellol ester for biomedical applications [25]. Nevertheless, the synthesis of these functional polymers requires non-renewable backbone chains (polyphosphazenes) to hold the citronellol chains. In addition, a chlorine-based hexachlorophosphazene is needed to synthesize polyphosphazenes [26]. Citronellyl, geranyl and neryl methacrylate monomers were also prepared by Worzakowska [27]. Branched polymers were obtained through the UV-photoinitiated polymerization of these functional methacrylated monomers at room temperature. Interestingly, among the polymers obtained, poly(citronellyl methacrylate) was the most thermally stable material [27].

**Scheme 1:**
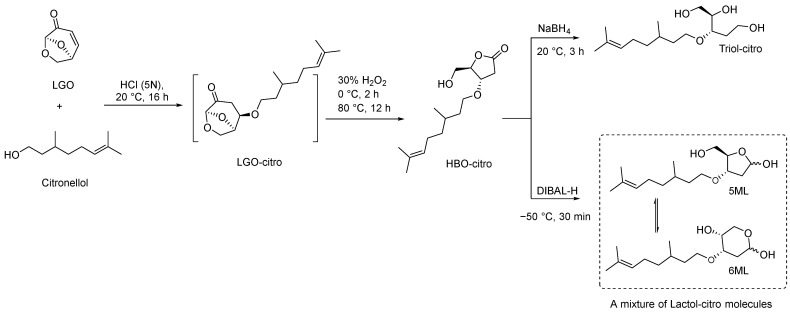
Recently reported syntheses of Triol-citro and Lactol-citro from LGO.

Given our strong expertise in levoglucosenone (LGO), a wood-based functional molecule produced at the scale of several tons per year [28,29,30], we have recently become interested in developing not only renewable functional monomers and polymers from LGO [31,32,33,34,35,36] but also biodegradation protocols to evaluate the end-of-life of our in-house corresponding materials. In this context, we recently reported the first fully renewable citronellol-containing monomers from LGO [34,35]. More precisely, we took advantage of the α,β-conjugated double bond of LGO to perform the acid-catalyzed [37] oxa-Michael addition of citronellol (Scheme 1). This step was followed by the in situ organic solvent-free H_2_O_2_-mediated Baeyer-Villiger oxidation to access HBO-citro. From the latter, fully renewable monomers (Triol-citro and Lactols-citro) were prepared through the selective reduction of the HBO lactone moiety. Notably, when NaBH_4_ was used as a reducing agent, it led to the formation of the tris-hydroxy monomer, Triol-citro. However, when diisobutylaluminum hydride (DIBAL-H) was adopted, a mixture of 5- and 6-membered cyclic forms of Lactol-citro (5ML and 6ML) were obtained (Scheme 1) [34]. Indeed, the ring-chain tautomerism of furanoses (5-membered rings) and pyranoses (6-membered rings) is very common in sugars [38]. These furanose-pyranose interconversions exist in equilibrium which led, in our case, to a mixture of Lactol-citro molecules. In our previous work [34], Triol-citro was selected to prepare renewable polyesters (P1–P4) via its polycondensation reaction with diacyl chlorides having different chain lengths (m = 1–4, respectively). When P1–P4 were exposed to Lipopan^®^ 50 BG, a commercial lipase from *Thermomyces lanuginosus*, an 80% degradation of P2–P4 was observed after 80 h [34]. Inspired by this work, we decided herein not only to prepare new fully renewable polyesters (P5–P8) that contain two types of lactol rings (5-membered and 6-membered) but also to study and compare the biodegradation of the newly synthesized lactol-based copolyesters (P5–P8) to that of the previously reported triol-based polymers (P1–P4). Furthermore, the equilibrium of 5ML and 6ML was studied by ^1^H NMR at three temperatures: 25 °C, 50 °C and 65 °C. 

## 2. Materials and Methods

Chemicals and reagents. Levoglucosenone was graciously provided by Circa group. Citronellol (Sigma Aldrich, Saint-Louis, MO, USA), diisobutylaluminum hydride (DIBAL-H) (Acros, Geel, Belgium), malonyl chloride 97% (Sigma Aldrich), succinyl chloride 95% (Sigma Aldrich), adipoyl chloride 98% (Sigma Aldrich), glutaryl chloride 97% (Sigma Aldrich), Lipopan^®^ 50 BG (Novozymes, Bagsværd, Denmark) a purified lipase from *Thermomyces lanuginosus* expressed in *Aspergillus oryzae*, were used as received. NMR solvents, including CDCl_3_ and DMSO-*d*_6_, were purchased from Cambridge Isotopes Laboratories. HPLC grade solvents were purchased from Thermofisher Scientific, Waltham, MA, USA, and used as received. Ultra-pure laboratory-grade water was obtained from MilliQ, 18.2 megaOhms. TLC analyses were performed on an aluminum strip coated with Silica Gel 60 F254 from Merck, revealed under UV-light (254 nm), then in the presence of potassium permanganate staining solution. All manipulations with air-sensitive chemicals and reagents were performed using standard Schlenk techniques on a dual-manifold line, on a high-vacuum line.

### 2.1. Characterization 

Nuclear Magnetic Resonance (NMR) spectroscopy. ^1^H NMR spectra were recorded on a Bruker Fourier 300 MHz (CDCl_3_ residual signal at 7.26 ppm and DMSO-*d*_6_ residual signal at 2.5 ppm). ^13^C NMR spectra were recorded on a Bruker Fourier 300 (75 MHz) (CDCl_3_ residual signal at 77.16 ppm and DMSO-*d*_6_ residual signal at 39.52 ppm). Data are reported as follows: chemical shift (δ ppm), multiplicity, coupling constant (*J* Hz), integral, assignment. All NMR assignments were also made using ^1^H-^1^H COSY, ^1^H-^13^C HMBC and ^1^H-^13^C HSQC spectra.

Size exclusion chromatography (SEC) was performed at 50 °C using an Agilent Technologies (Santa Clara, CA, USA) 1260 Infinity Series liquid chromatography system with an internal differential refractive index detector, a viscometer detector, a laser and two PLgel columns (5 µm MIXED-D 300 × 7.5 mm), using 10 mM Lithium Bromide in HPLC grade dimethylformamide as the mobile phase at a flow rate of 1.0 mL/min. Calibration was performed with poly (methyl methacrylate) standards from Agilent Technologies. Typically, ~3 mg of each sample was dissolved in 1 mL of DMF (10 mM LiBr) prior to analysis.

Thermogravimetric Analysis (TGA) was measured with a TGA Q500 (TA Instruments, New Castle, DE, USA). Typically, ~2 mg of each sample was equilibrated at 50 °C for 30 min and was flushed with highly pure nitrogen gas. All the experiments were performed with a heating rate of 10 °C/min up to 500 °C. The reported values *T*_d5%_ and *T*_d50%_ represent the temperature at which 5% and 50% of the mass is lost, respectively. 

Differential Scanning Calorimetry (DSC) was performed with a DSC Q20 (TA Instruments). Typically, ~8 mg sample was placed in a sealed pan, flushed with highly pure nitrogen gas and passed through a heat-cool-heat cycle at 10 °C/min in a temperature range of −80 °C to 100 °C. Three heat/cool cycles were done for each sample where the last two cycles were dedicated to analyzing the heat flow of the sample after being cooled in controlled conditions. The *T*_g_ values recorded in this work are those obtained from the third cycle.

Fourier-transform infrared spectroscopy (FTIR) was recorded on a Cary 630 FTIR Spectrometer by Agilent (Wilmington, DE, USA).

### 2.2. Synthesis of Monomers

**Scheme 2:**
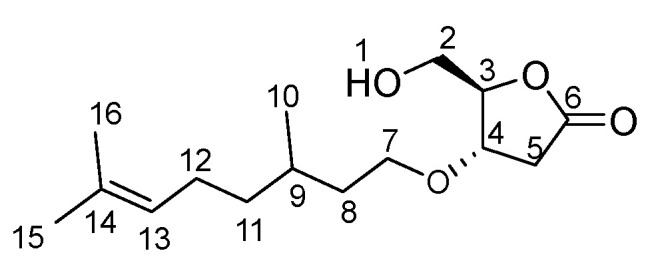
Structure of HBO-citro.

HBO-citro (Scheme 2). A biphasic mixture of LGO (50 g, 0.4 mol), citronellol (512 mL, 1.8 mol) and HCl (5 N, 0.6 mol) was stirred at room temperature for 16 h. The resulting mixture was cooled down with an ice bath followed by the dropwise addition of a 30% solution of H_2_O_2_ (2 mL) for 2 h. After completion of the addition, the reaction was heated up to 80 °C and stirred for 12 h. The presence of H_2_O_2_ was evaluated with peroxide strips and any residual H_2_O_2_ was quenched using sodium sulfite. The reaction was extracted with ethyl acetate (two times). Organic layers were washed with brine, dried over anhydrous MgSO_4_, filtered and evaporated to dryness. This step was followed by distillation to remove excess citronellol. The crude product after distillation was purified by flash chromatography (gradient 90/10 to 20/80, cyclohexane/ethyl acetate as eluant) to give 62 g of HBO-citro as a pale-yellow oil (58%). 

^1^H NMR (CDCl_3_), δ_H_: 5.01 (broad t, *J* = 5.19 Hz, 1H, H_13_), 4.43 (s, 1H, H_3_), 4.11 (d, *J* = 7.0 Hz, 1H, OH), 3.84 (dd, *J* = 3.3 and 12.4 Hz, 1H, H_4_), 3.65 (dd, *J* = 3.3 and 12.4 Hz, 2H, H_2_), 3.39 (m, 2H, H_7_), 2.81 (dd, *J* = 7.0 and 18.1 Hz, 1H, H_5a_), 2.44 (dd, *J* = 3.3 and 18.1 Hz, 1H, H_5s_), 1.90 (m, 2H, H_12_), 1.61 (s, 3H, H_15_), 1.53 (s, 3H, H_16_), 1.27-1.07 (m, 4H, H_11_, H_8_), 0.82 (d, *J* = 6.4 Hz, 3H, H_10_).

^13^C NMR (CDCl_3_), δ_C_: 176.7 (C_6_), 131.2 (C_14_), 124.6 (C_13_), 85.9 (C_3_), 76.1 (C_4_), 67.6 (C_7_), 62.1 (C_2_), 37.1 (C_11_), 36.5 (C_8_), 35.9 (C_5_), 29.3 (C_9_), 25.7 (C_15_), 25.3 (C_12_), 19.4 (C_10_) 17.6 (C_16_).

**Scheme 3:**
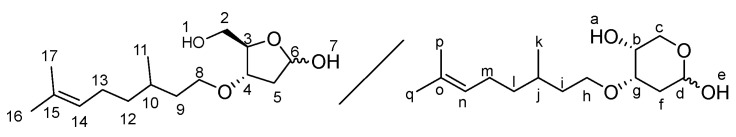
Structures of 5ML (**left**) and 6ML (**right**).

Lactol-citro (Scheme 3). A 1.2 M solution of DIBAL-H (27.5 mL of DCM, 33 mmol) was added dropwise to a solution of HBO-citro (4.5 g, 15 mmol) in DCM (68 mL) at −50 °C. The reaction mixture was stirred for 30 min at −50 °C then quenched with a 20% aqueous solution of citric acid (30 mL). The reaction was warmed to room temperature then stirred until the aluminum salt disappeared. Layers were separated and the organic layer was washed with brine, dried over anhydrous magnesium sulfate, filtered and evaporated to dryness. The crude product was purified by flash chromatography (gradient 90/10 to 20/80, cyclohexane/ethyl acetate as eluant) to give 3.63 g of Lactol-citro molecules as a colorless oil (89%). 

The full interpretation of ^1^H and ^13^C NMR (DMSO-*d*_6_) is provided in Figures S1–S5 in the Supporting Information (SI).

### 2.3. Synthesis of Polymers

**Scheme 4:**
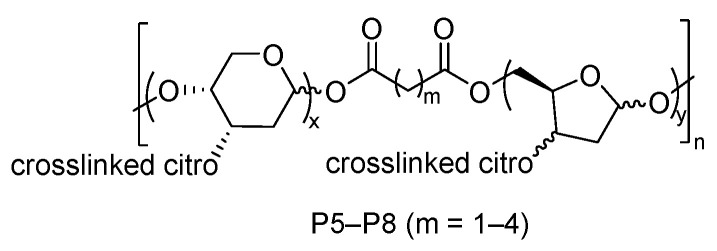
Structure of P5–P8 obtained from the polycondensation of 5ML, 6ML and diacyl chlorides.

P5–P8 (Scheme 4). A typical two-step melt polycondensation experiment (run 1, Table 1) was performed as follows. Under N_2_ atmosphere, Lactol-citro (500 mg) was charged into a 10 mL round bottom flask connected to a vacuum line, equipped with a condensate trap. The flask was cooled down with an ice bath, then 1 equiv. of malonyl chloride (0.18 mL) was added. The mixture was stirred at room temperature for 19 h. The temperature was then increased gradually up to 50 °C. A high vacuum (10^−3^ bar) was then applied for 3 h. Additional polyesters were prepared with this protocol, employing other diacyl chlorides (succinyl chloride, adipoyl chloride and glutaryl chloride).

^1^H and ^13^C NMR (DMSO-*d*_6_) of P5–P8 are provided in Figures S6–S13 in the Supporting Information (SI).

### 2.4. Enzymatic Degradation

Before its use, the activity of the enzyme was assessed using the *p*-nitrophenyl butyrate colorimetric assay. Absorbance was monitored at 400 nm and the results were compared with a *p*-nitrophenol calibration curve. P5–P8 were ground using a spatula to yield powdered polyester samples. 50 mg of the ground samples were placed in 4 sealed vials containing phosphate buffer (3 mL, 0.05 M) and 10 mg/mL of Lipopan^®^ 50 BG (50 KLU/g). Lipopan^®^ 50 BG contains lipases from *Thermomyces lanuginosus* expressed in *Aspergillus oryzae* commercially used in bakery. The enzyme concentration was adapted from Alejandra et al. [39] The mixtures were then incubated at the pH and temperature optima for the enzyme (37 °C, pH 7) and stirred gently at 50 rpm for 48 h. The reactions were then stopped by immersing the tubes in an ice bath. To remove the enzyme, the hydrolyzed polymers were washed three times with 10 mL of water followed by centrifugation for 10 min at 10 °C and 4750 rpm. The resulting products were freeze-dried to remove all traces of water before being subjected to mass loss measurement and characterization of polyester enzymatic degradation. All isolated powders were analyzed by ^1^H NMR, SEC, DSC and FTIR. Controls were realized for each polymer in phosphate buffer solution without enzyme and showed no degradation.

## 3. Results and Discussion

### 3.1. Lactol-Citro

**Figure 1:**
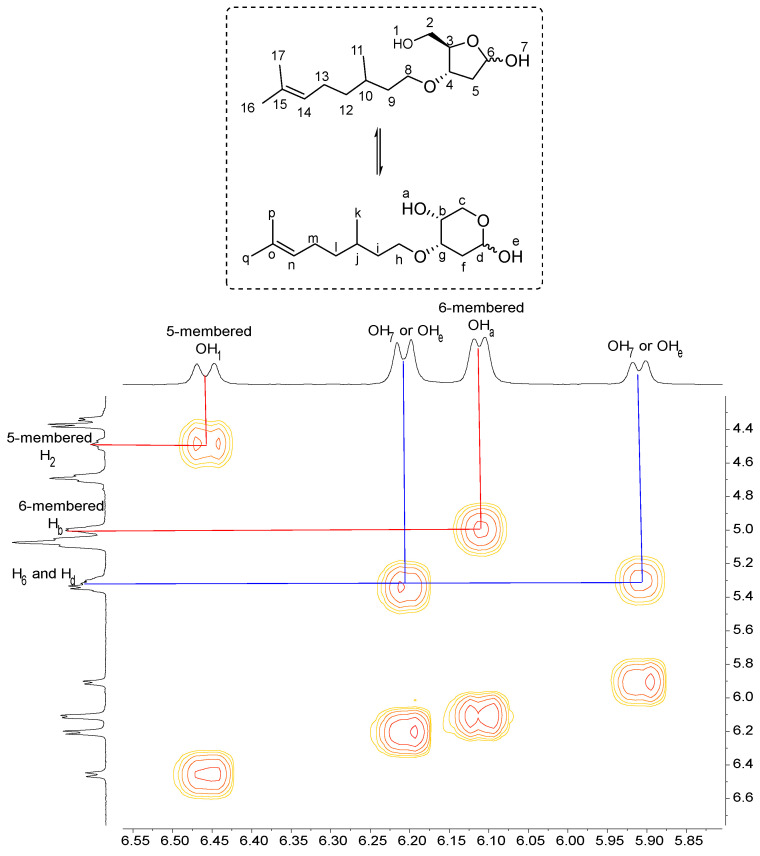
^1^H-^1^H COSY (DMSO-d_6_) spectrum of 5ML and 6ML mixture.

**Figure 2:**
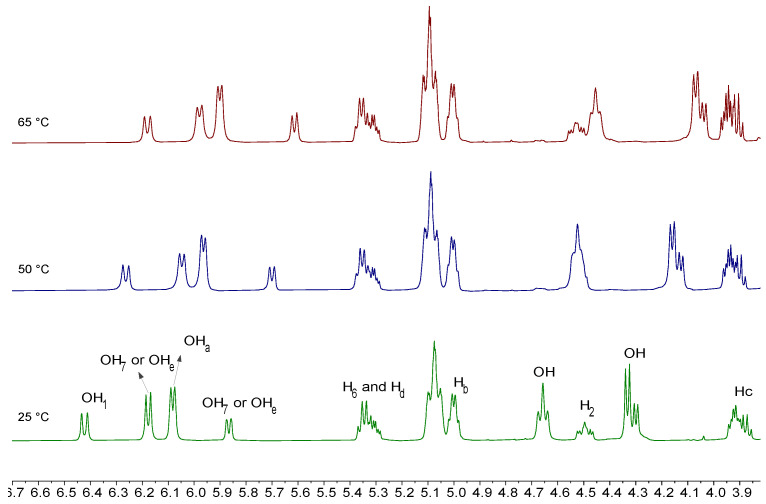
Effect of temperature on the ^1^H NMR (DMSO-d_6_) of 5ML ⇌ 6ML interconversion.

The synthesis of 5- and 6-membered Lactol-citro (5ML and 6ML, respectively) was performed from HBO-citro according to a recently reported procedure [34]. It is worth mentioning that although the reduction of HBO-ctiro is easy to perform, reaction conditions must be strictly controlled to avoid the formation of Triol-citro (Scheme 1). Anhydrous solvents, a nitrogen atmosphere and a short time period (30 min) are all necessary to promote the complete conversion of HBO-citro to Lactol-citro. Otherwise, in the presence of a trace amount of water, and for durations longer than 30 min, Triol-citro was observed. Although the separation of the two products was not possible, two cyclic structures of Lactol-citro were easily distinguishable by ^1^H-^1^H COSY NMR (Figure 1). The major occurrence (65%) of the more stable 6-membered derivative was observed at 6.11 ppm, corresponding to OH_a_ group of 6ML. On the other hand, the proton resonance of OH_1_ at 6.46 ppm identified the percentage of the less stable 5-membered lactol cycle, which was interestingly found to be 35%. Since the polycondensation using these molecules was performed in two steps (room temperature under N_2_ and 50 °C under vacuum, vide infra), we decided to study equilibrium of 5ML ⇌ 6ML interconversions at different temperatures (25 °C, 50 °C and 65 °C) using ^1^H NMR. The percentages of each of the 5ML and 6ML were monitored using the hydroxyl resonances OH_1_ and H_2_, and OH_a_ and H_c_, respectively. Figure 2 shows that temperature has an impact on the chemical shifts of the aforementioned protons; mainly on the hydroxyl peaks that showed an up-field shifting (to the right) when the temperature was increased from 25 °C to 50 °C and 65 °C. For example, at 25 °C, the chemical shifts of OH_1_ and OH_a_ are 6.42 ppm and 6.09 ppm, respectively. However, when the NMR measurement was performed at 65 °C, a shift of these two signals to 6.18 ppm and 5.90 ppm was recorded. Interestingly, when integrating the peaks of H_2_ and H_c_ we found a 3% and 9% increase of 6ML at 50 °C and 65 °C, respectively. Indeed, the variation of the 5ML:6ML ratio must be finely monitored during the two polymerization steps (vide infra)—especially in the presence of highly reactive co-monomers, such as acyl chloride—to allow the controlled incorporation of the Lactol-citro along the polyester chain. For these reasons, it is more convenient to carry out the polymerization step at 50 °C where only 3%, towards the formation of the more stable pyranose-like derivative, was obtained. 

### 3.2. Polycondensation

The potential of Lactol-citro (5ML–6ML) was then investigated in the synthesis of new renewable functional polyesters. Solvent-free polycondensations were first performed using aliphatic diacyl chlorides as co-monomers with different chain lengths (m = 1–4) (Table 1). The first step of the polycondensation was started at room temperature for 19 h. The temperature was then increased to 50 °C to allow the polymerization of the oligomers formed in the 1st step while also controlling the interconversion of 5ML–6ML. The products isolated in both steps were found to be insoluble in all classical solvents (e.g., tetrahydrofuran, dioxane, chloroform), except in dimethylformamide (DMF) and dimethyl sulfoxide (DMSO), where partial solubility was observed. All our attempts to enhance the solubility of the prepared polyesters were unsuccessful. For example, we tried to use polymerization methods with solvents (e.g., THF) and base (e.g., pyridine) to control the condensation reactions of the first step or after oligomer formation. Furthermore, different times and temperatures were tested; however, only partially or non-soluble polymers could be isolated. Nevertheless, such descent solubility was sufficient to characterize the polymers (vide infra).

The NMR spectra assignment was consistent with the formation of cross-linked copolyesters. First, after the incorporation of acyl chloride co-monomer, the signals of the free hydroxyl groups of 5ML and 6ML in the range of 6.47–5.90 ppm and at 4.96 and 4.38 ppm disappeared (SI, Figures S6–S13). Also, the carbonyl groups of the polyesters formed were clearly detected by ^13^C NMR in the region of 175.0–169.0 ppm (SI, Figures S7, S9, S11 and S13 for P5, P6, P7 and P8 respectively). On the other hand, no presence of H_14_ and H_m_ of the citronellol double bond at 5.07 ppm was observed, demonstrating that the pendent citronellol moieties were possibly cross-linked at a relatively elevated temperature (50 °C) or/and in the presence of diacyl chloride. The crosslinking was more obvious when analyzing the ^13^C NMR spectra that showed the complete disappearance of C_15_, C_o_ and C_14_, C_n_ signals at 130.9 and 125.0 ppm, respectively. Indeed, this thermally-induced and/or acid-catalyzed complete crosslinking was observed regardless of the co-monomer chain length (acyl chlorides, m = 1–4). FTIR analysis of P5–P8 was performed and showed the disappearance of the OH band of the lactol monomers at 3400 cm^−1^ as well as the appearance of a carboxyl group band at 1730 cm^−1^, which corresponds to the newly formed esters (SI, Figure S15).

Size exclusion chromatography (SEC) was used to measure the number-average molecular weight (*M*_n_) as well as the dispersity (*Đ*). The polymers were partially soluble, or not soluble, in DMF prior to SEC analysis; thus, the results obtained by SEC represent only the soluble portions of the polymers. The copolyesters obtained from the lactol derivatives showed a higher *M*_n_ than the recently reported Triol-citro-based branched polyesters (P1–P4), e.g., 25.8 kDa for P8 (run 4, Table 1) vs. 1.6 kDa for the polyester of Triol-citro and glutaryl chloride (P4). Indeed, this is due to the formation of more complex and less soluble cross-linked/branched structures when Triol-citro, which contains three hydroxy groups with different reactivity, was employed.

**Figure 3:**
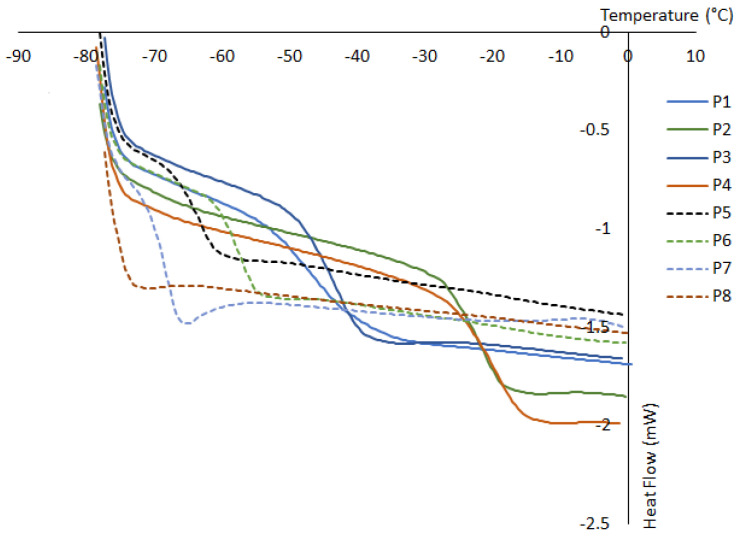
DSC thermograms of P1–P8.

The thermal analyses of P5–P8 were examined using differential scanning calorimetry (DSC) and thermogravimetric analysis (TGA) (SI, Figures S20–S22 and S27–S30, respectively). DSC showed negative glass transition (*T*_g_) values for all polymers. Nevertheless, it is worth mentioning that P5–P8 showed higher *T*_g_ (−57 °C to −67 °C) than polyesters prepared using Triol-citro [34] (Figure 3). This confirms the higher mobility/flexibility of the chains contained within the Lactol-citro based structures. Having *T*_g_ values in this negative range is attractive for applications that require a *T*_g_ below body temperature, such as in the biomedical field. No melting temperature (*T*_m_) was observed for any of the polymers reported in this study. Moreover, no effect of the chain length of the acyl chloride was detected. This is probably due to the effect of the citronellol moiety which outweighs that of the number of carbons in the aliphatic co-monomer unit. This is in agreement with the formation of a more stable branched structure when the Triol-citro was engaged [34]. TGA showed that P5–P8 have lower thermal resistance than P1–P4. For example, P8 exhibited a *T*_d5%_ of 127 °C and a *T*_d50%_ of 210 °C, while those of Triol-citro polymer (P4) were 170 and 386 °C, respectively.

### 3.3. Enzymatic Degradation

**Figure 4:**
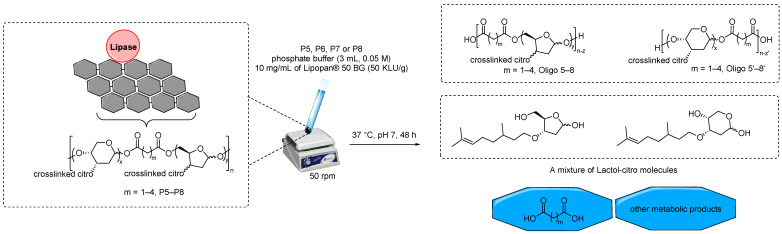
Illustration of the enzymatic degradation of P5–P8 and the corresponding hydrolyzed products.

Given the promising degradation behavior that we have recently seen for the Triol-based polyesters (P2–P4) [34], we decided to investigate the enzymatic degradation of P5–P8 following the same procedure in the presence of a commercial lipase enzyme from *Thermomyces lanuginosus* (Lipopan^®^ 50 BG, Novozymes^®^, Bagsværd, Denmark). Indeed, a lipase-mediated degradation occurs first by a surface erosion process triggered by the adsorption of the large-sized enzyme on the polymer surface. This step is followed by the hydrolysis of the ester bonds to form the corresponding oligomers with terminal hydroxyl and carboxylic acid. In this study, we were able to monitor the hydrolysis of the polymers that occurred during the second stage of the degradation process. Specifically, the hydrolysis of the ester bonds led to the formation of shorter oligomers and/or constituting monomers (Figure 4), the existence of which can be easily monitored by ^1^H NMR and FTIR, as well as by SEC and DSC. Comparison of the initial analyses of the P5–P8 polymers in Table 1 with the results obtained after treatment with Lipopan^®^ 50 BG (Table 2), demonstrates the effective enzymatic degradation of the copolymers. It is worth mentioning that purification of the degraded cross-linked oligomers and (co-)monomers by simple methods, such as precipitation, was not possible. Nevertheless, due to the presence of hydroxy and carboxylic acid groups in the products after enzymatic degradation (vide infra), they can be engaged as a whole in polycondensation reactions; however, this would lead to the formation of several cross-linked oligomers/polymers with no control of molecular weight or dispersity.

Due to the low solubility of P5–P8, all our attempts to make films by solvent casting failed. Thus, we studied the biodegradation using their powder form, which was collected by grinding at the end of the polymerization reactions. Notably, P5–P8 were degraded at a significantly higher rate (∼80% mass loss in 48 h) compared to Triol-citro based polyesters P2–P4 (∼80% mass loss in 96 h). Indeed, a highly cross-linked/branched structure as in P1–P4, can induce a globular polymer conformation that hinders the accessibility of the enzyme to the inner surface of the chains and delays/prevents the hydrolysis rate of the ester bonds. Therefore, in contrast to the polyester of Triol-citro and malonyl chloride (P1), a successful degradation was observed in the case of P5, as evidenced by the appearance of hydroxyl vibration band by FTIR. Indeed, the hydrolytic step of enzymatic degradation is determined by the hydrolysis time for polymers to form into shorter chains and the diffusion of water through the material, which can be slowed down in the case of a short aliphatic co-monomer chain, such as that of malonyl chloride (m = 1) accompanied by more complex cross-linked structures, as in the case of P1. 

**Figure 5:**
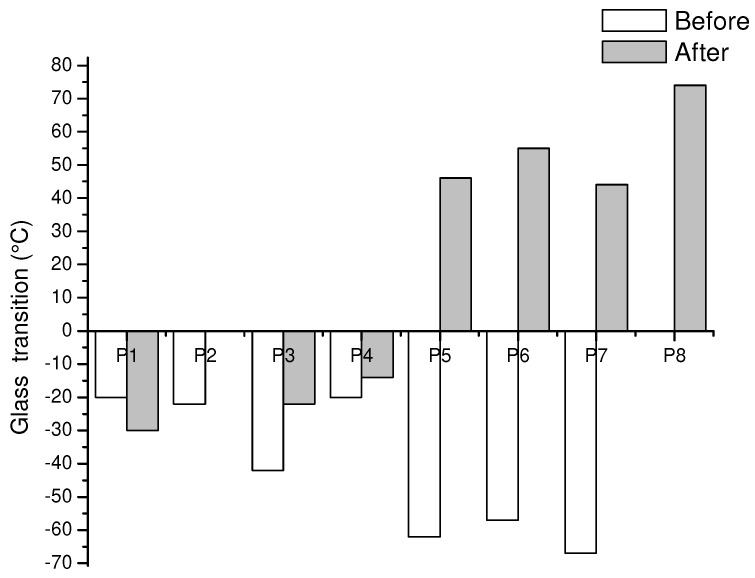
Glass transition temperature (*T*_g_) of P1–P8 before and after enzymatic degradation (No *T*_g_ was observed for P8 and P2 before and after degradation, respectively).

Examination of the ^1^H NMR spectra showed the appearance of new peaks in the region of 5.53–4.59 ppm, which correspond to the hydroxyl protons of the hydrolyzed products (SI, Figure S14). In agreement with the ^1^H NMR of P5–P8, FTIR showed the appearance of a large hydroxyl vibration band at around 3350 cm^−1^ after enzymatic degradation (SI, Figure S16). The enzymatic degradation was also checked using SEC to determine the reduction in *M*_n_ (SI, Figures S31–S34). Notably, SEC and DSC also showed a considerable degradation of P5–P8. For example, a significant decrease in *M*_n_ of P7 from 12.5 kDa (run 7, Table 1) to 0.8 kDa (run 3, Table 2) was observed. The thermal properties of the resulting hydrolyzed products were also analyzed by DSC (SI, Figures S23–S26). A great variation of *T*_g_ from −67 °C to 44 °C (Δ*T*_g_ = 111 °C) was registered. Notably, Δ*T*_g_ of P5–P8 was found to be much larger than those recorded for P1–P4 (see Figure 5 for more information). Such an increase in *T*_g_ can be due to a greater number of polymer chain cleavages that lead to lesser chain entanglement, and in turn a higher degree of crystallinity. The increase of crystalline portions within the oligomer chains can reduce molecular mobility, which causes an increase in *T*_g_ and, in turn, Δ*T*_g_. 

## 4. Conclusions

Ring-chain tautomerism of furanoses (5-membered rings) and pyranoses (6-membered rings) is very common in sugar chemistry. LGO was used as a starting material to produce new renewable 5- and 6-membered Lactol-citro molecules (5ML and 6ML) with citronellol side chains. The interconversion of both molecules was studied at a temperature ranging from 25 °C to 65 °C and showed the expected higher occurrence of the more stable cycle (6ML) at higher temperatures.

The polycondensation of the Lactol-citro mixture and aliphatic diacyl chlorides was then performed in solvent- and catalyst-free conditions. New LGO-derived copolyesters, with low *T*_g_ ranging from −57 °C to −67 °C, were formed. Furthermore, the analyses showed that the citronellol chains were unexpectedly cross-linked during polymerization. The enzymatic degradation of the polyesters, P5–P8, was then investigated in the presence of Lipopan^®^ 50 BG under the same conditions recently reported for P1–P4 polymers obtained with Triol-citro and diacyl chlorides (m = 1–4). ^1^H NMR, FTIR, SEC and DSC analyses showed a greater tendency to hydrolyze P5–P8 than P1–P4 when a lipase is used. This was probably due to the formation of a less strained structure when 5ML and 6ML were engaged, rather than Triol-citro monomer.

## Figures and Tables

**Table 1:** Two-step polycondensation of 5ML and 6ML with acyl chloride derivatives.

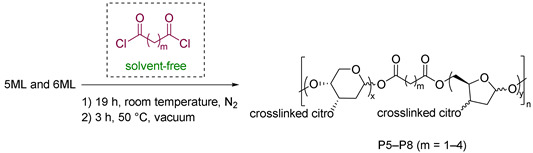					
Run	Polymer	*M*_n_ (kDa) ^1^	*Đ* ^1^	*T*_g_ (°C) ^2^	*T*_d__50%_ (°C) ^3^
1	P5	- ^4^	-	−62	198
2	P6	- ^4^	-	−57	205
3	P7	12.5	1.3	−67	243
4	P8	25.8	1.8	-	210

^1^ Determined in DMF (10 mM LiBr) at 50 °C, only showed the soluble parts of the polymers. ^2^ Glass transition determined by DSC. ^3^ TGA degradation temperature at which 50% (*T*_d50%_) mass loss was observed under nitrogen. ^4^ Completely insoluble in DMF.

**Table 2:** Characterization of P5-P8 after enzymatic degradation using Lipopan^®^ 50 BG.

Run ^1^	Polymer	*M*_n_ (kDa) ^2^	*T*_g_ (°C) ^3^	Δ*T*_g_ (°C) ^4^	^1^H NMR ^5^	FTIR ^6^
1	P5	0.7	46	108	✓	✓
2	P6	0.8	55	112	✓	✓
3	P7	0.8	44	111	✓	✓
4	P8	0.8	74	- ^7^	✓	✓

^1^ Runs 1–4 are listed, following the same order as Table 1. ^2^ Determined in DMF (10 mM LiBr) at 50 °C. ^3^ Glass transition determined by DSC, temperature ramp 10 °C/min. ^4^ Δ*T*_g_ = *T*_g_(after degradation) −*T*_g_(before degradation). ^5 1^H NMR of the hydrolyzed product in DMSO-*d*_6_, ✓: presence of hydroxyl signals. ^6^ Infra-red of the hydrolyzed product, ✓: presence of hydroxyl band. ^7^ No *T*_g_ was observed for P8 before degradation.

## Data Availability

Not applicable.

## Supplementary Materials

The following supporting information can be downloaded at: https://www.mdpi.com/article/10.3390/polym14102082/s1, NMR (Figures S1–S14), FTIR (Figures S15–S19), DSC (Figures S20–S26), TGA (Figures S27–S30) and SEC (Figures S31–S34).
