# Supplementary material for: Synthesis and Enzymatic Degradation of Sustainable Levoglucosenone-Derived Copolyesters with Renewable Citronellol Side Chains
Source: Polymers (Basel). 2022 May 20;14(10):2082. doi: 10.3390/polym14102082 (PMC9146931; doi:10.3390/polym14102082)
Supplement: Supplementary file 1 [file polymers-14-02082-s001.zip › polymers-1715111-supplementary.pdf]

Supporting Information

Synthesis and Enzymatic Degradation of Sustainable Levoglucosenone-derived Copolyesters with Renewable Citronellol Side Chains

Sami Fadlallah *, Quentin Carboué *, Louis M. M. Mouterde, Aihemaiti Kayishaer, Yasmine Werghi, Aurélien A. M. Peru, Michel Lopez and Florent Allais *

URD Agro-Biotechnologies Industrielles (ABI), Centre Européen de Biotechnologie et de Bioéconomie (CEBB), AgroParisTech, 51110 Pomacle, France; louis.mouterde@agroparistech.fr (L.M.M.M.); aihemaiti.kayishaer@gmail.com (A.K.); yasmine.werghi1@gmail.com (Y.W.); aurelien.peru@agroparistech.fr (A.A.M.P.); michel.lopez@agroparistech.fr (M.L.)

* Correspondence: sami.fadlallah@agroparistech.fr (S.F.); quentin.carboue@agroparistech.fr (Q.C.); florent.allais@agroparistech.fr (F.A.)

Table of contents

NMR 2

FTIR 13

DSC 15

TGA 20

SEC 23

NMR

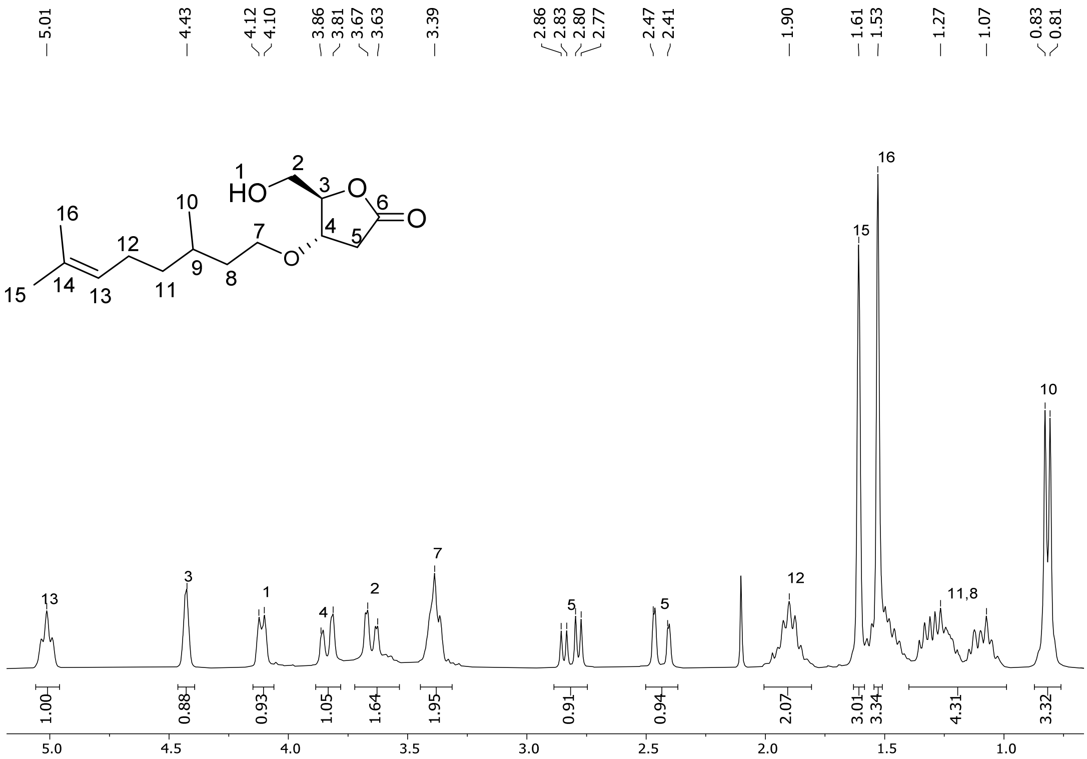

Figure S1. ^{1}H NMR ($CDCl_3$) spectrum of HBO-citro.

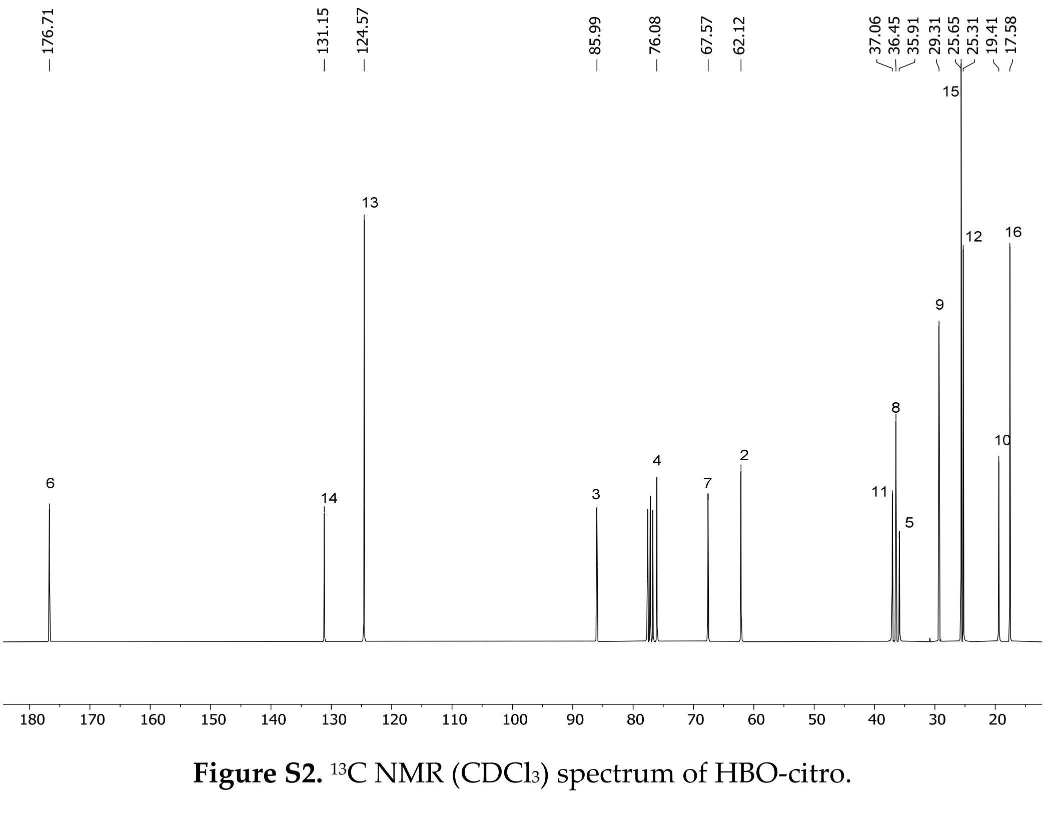

Figure S2. ^{13}C NMR ($CDCl_3$) spectrum of HBO-citro.

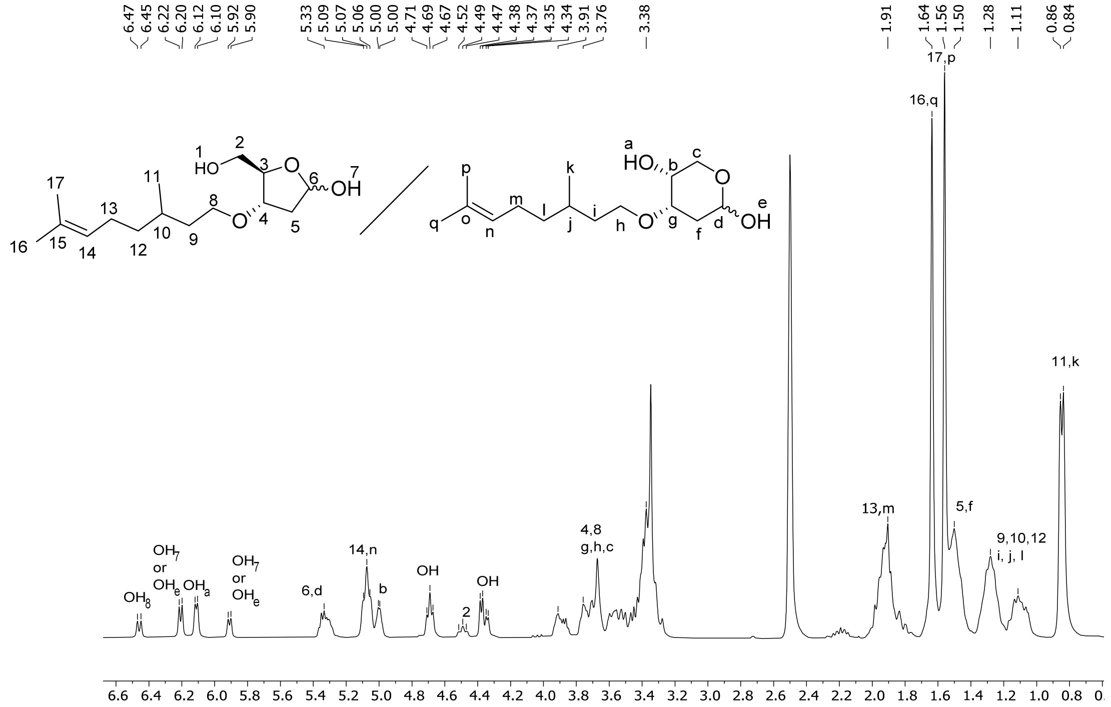

Figure S3. ^{1}H NMR (DMSO-d_6) spectrum of Lactol-citro molecules.

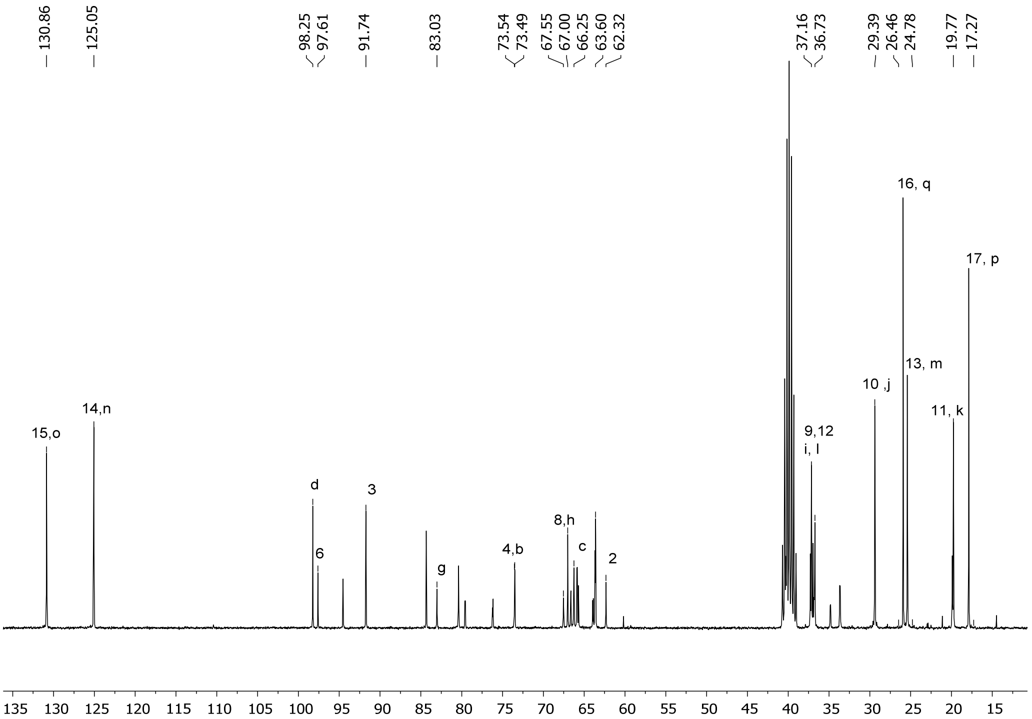

Figure S4. ^{13}C NMR (DMSO-d_6) spectrum of Lactol-citro molecules.

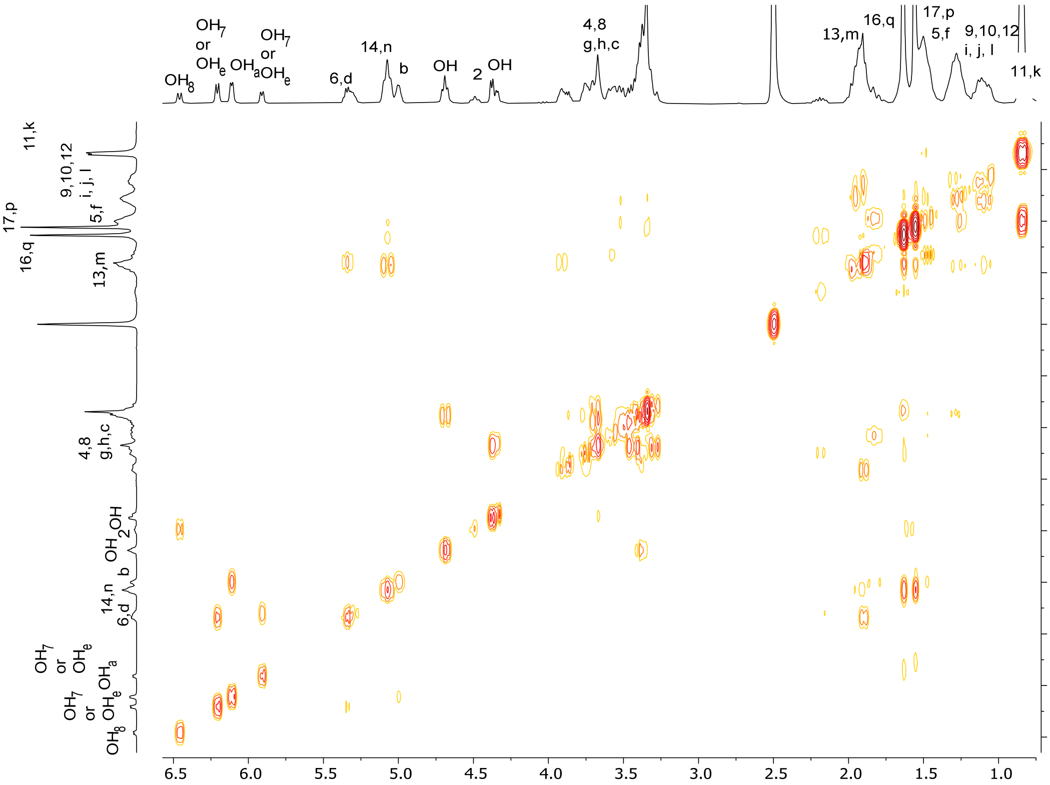

Figure S5. ^{1}H-^{1}H COSY (DMSO-d_6) spectrum of Lactol-citro molecules.

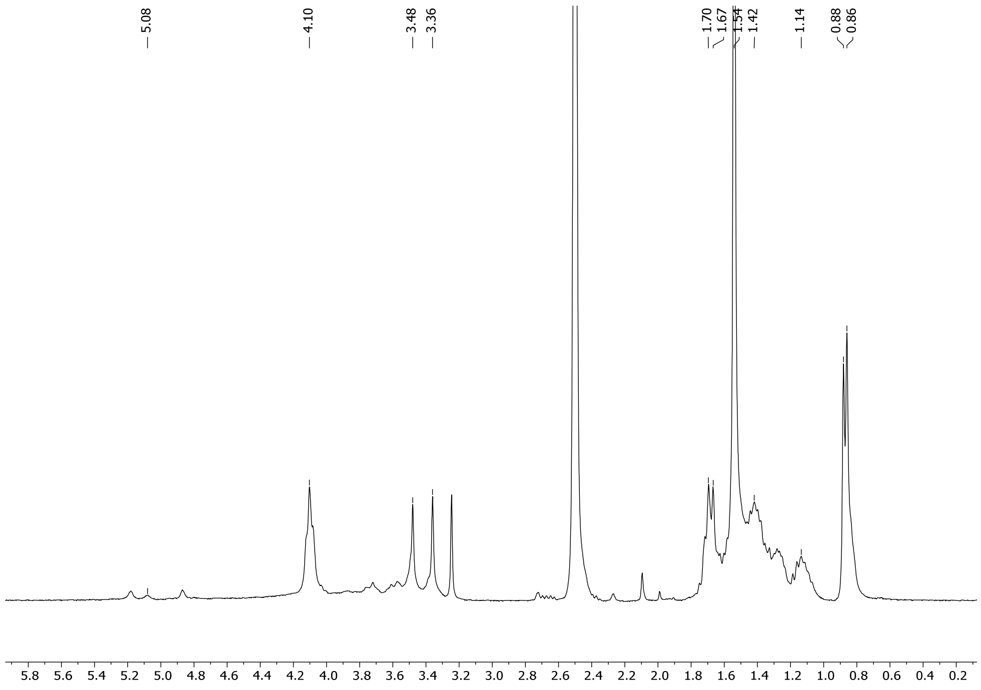

Figure S6. Typical ^{1}H NMR (DMSO-d_6) spectrum of P5.

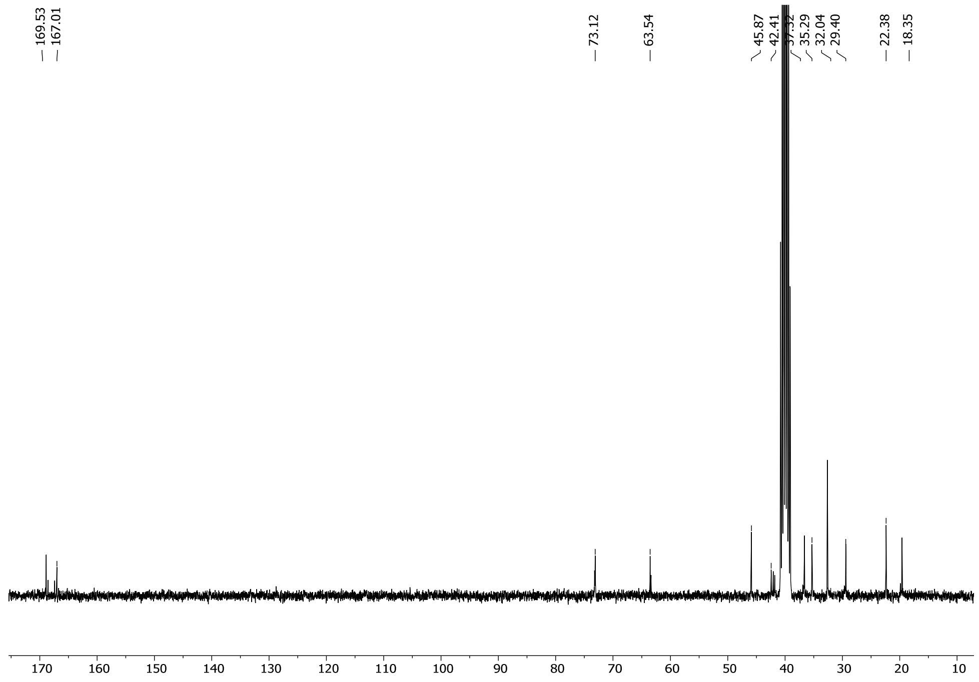

Figure S7. Typical ^{13}C NMR (DMSO-d_6) spectrum of P5.

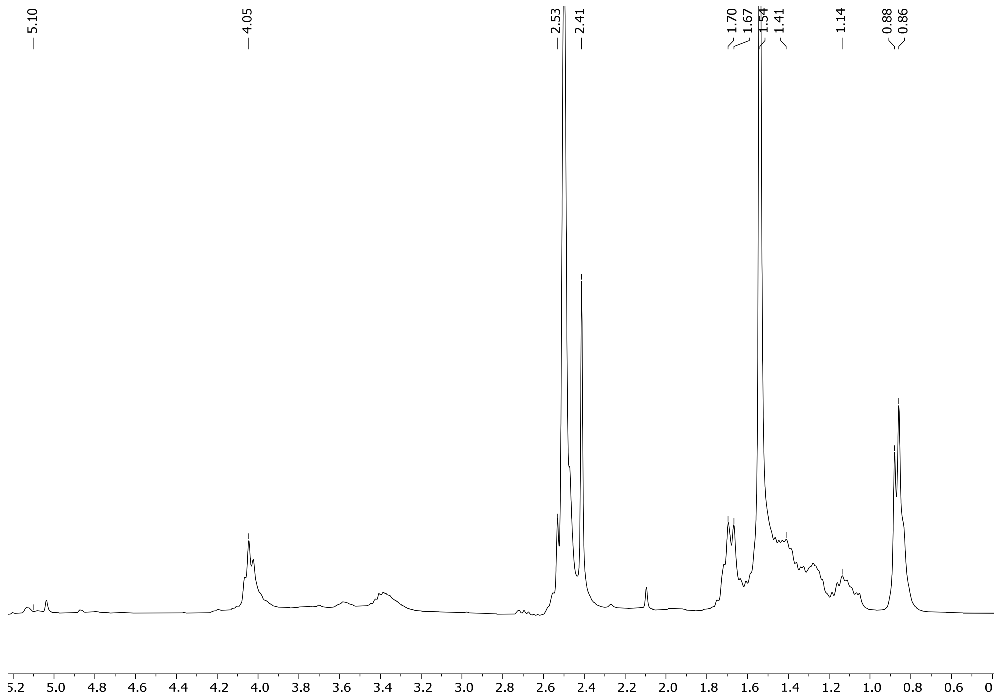

Figure S8. Typical ^{1}H NMR (DMSO-d_6) spectrum of P6.

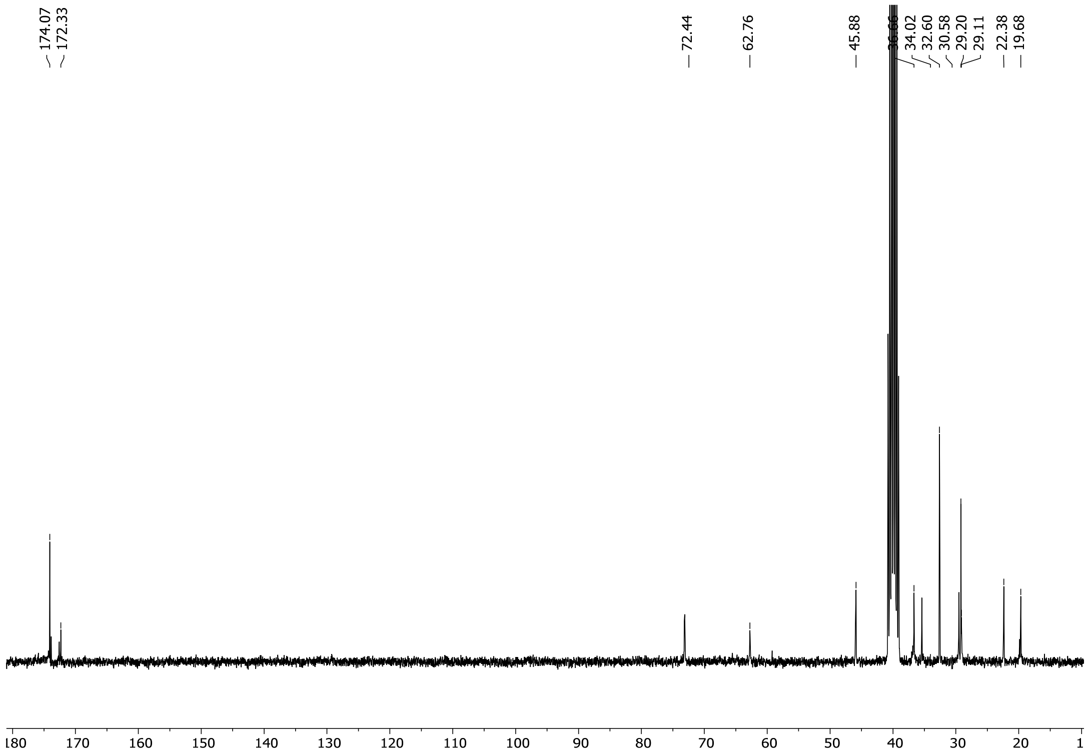

Figure S9. Typical ^{13}C NMR (DMSO-d_6) spectrum of P6.

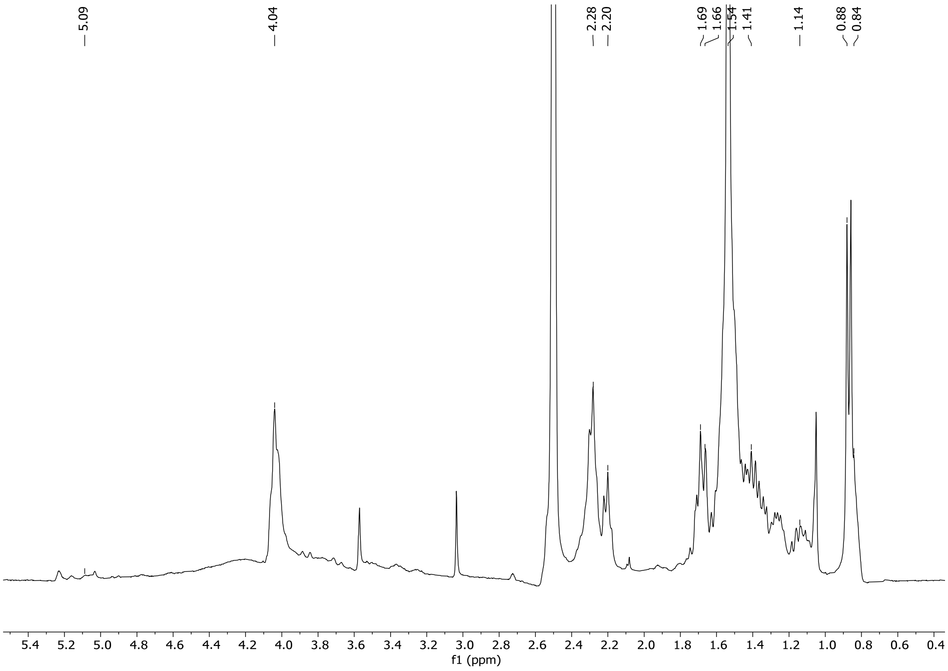

Figure S10. Typical ^{1}H NMR (DMSO-d_6) spectrum of P7.

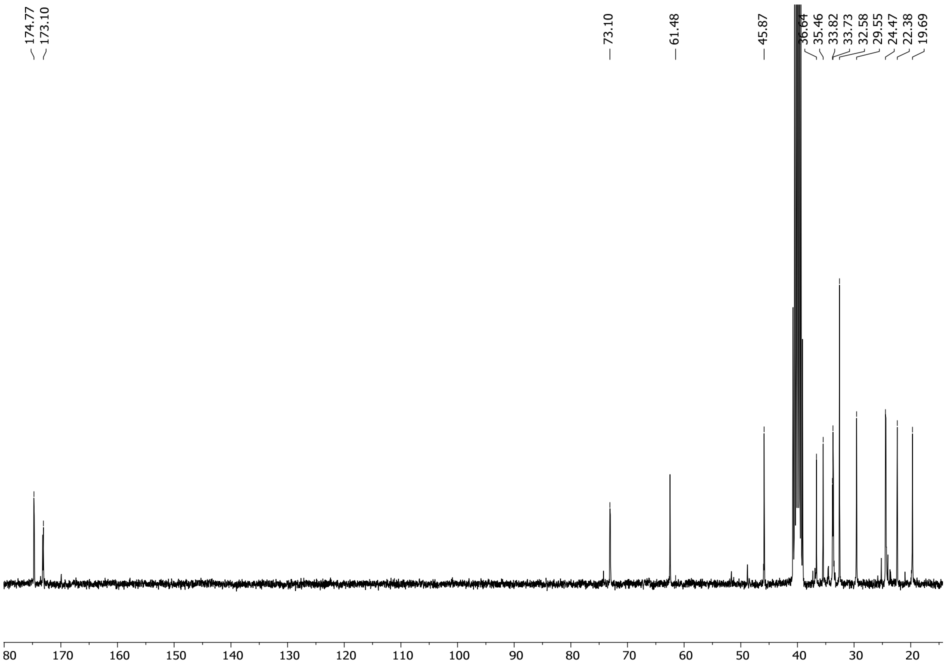

Figure S11. Typical ^{13}C NMR (DMSO-d_6) spectrum of P7.

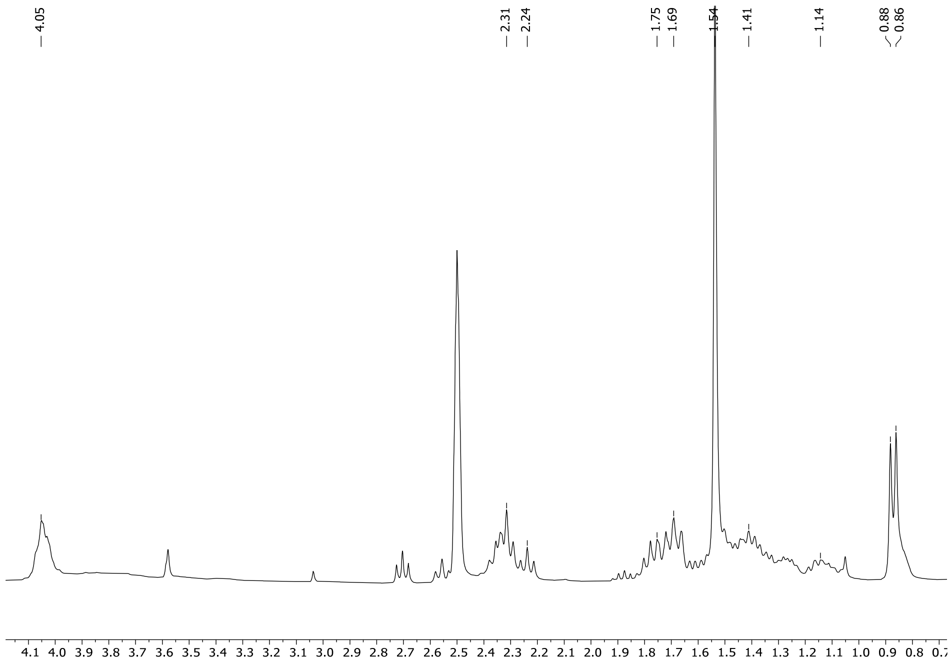

Figure S12. Typical ^{1}H NMR (DMSO-d_6) spectrum of P8.

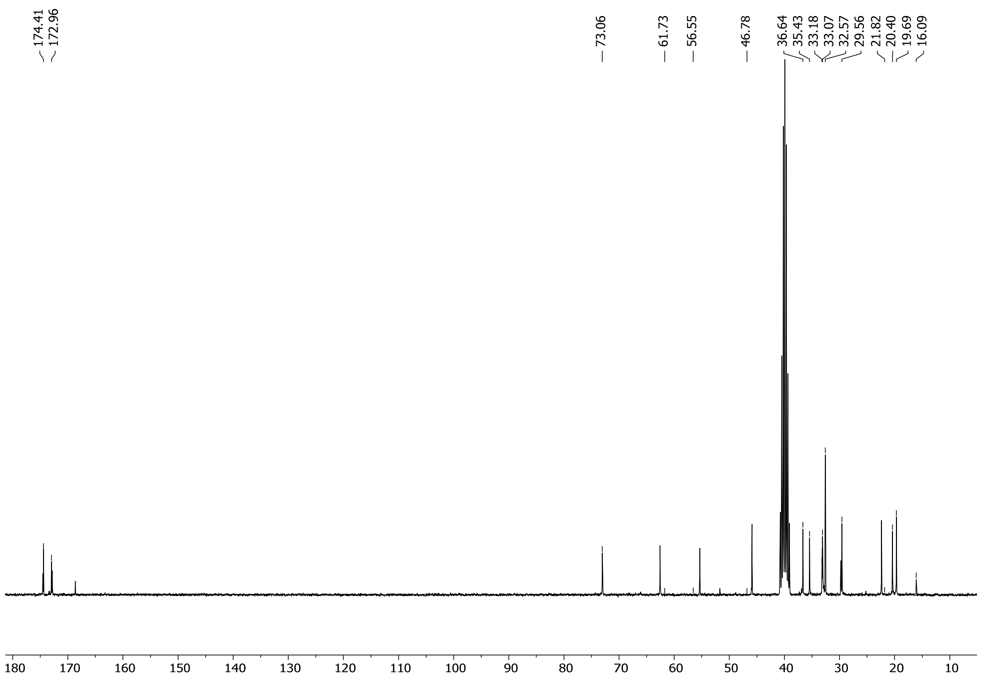

Figure S13. Typical ^{13}C NMR (DMSO-d_6) spectrum of P8.

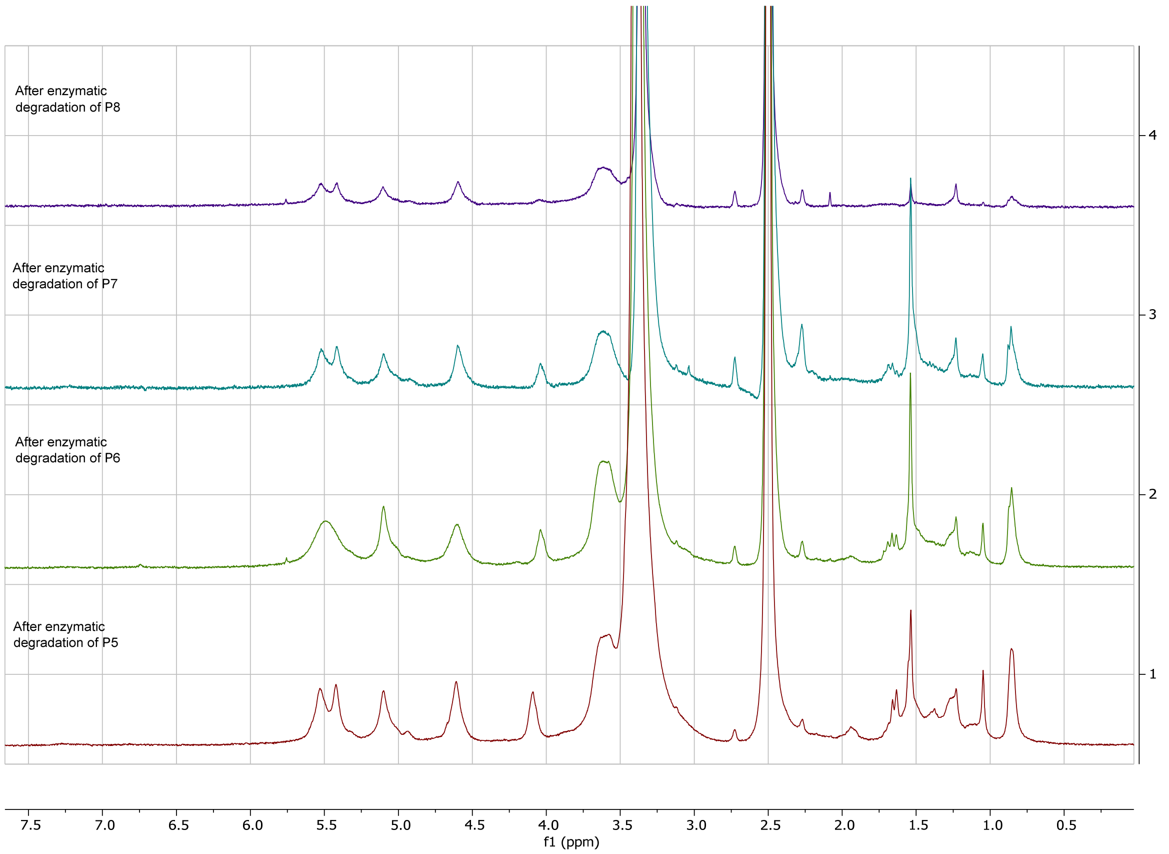

Figure S14. ^1H NMR (DMSO-d_6) spectra of the hydrolyzed products of P5, P6, P7 and P8 after enzymatic degradation (spectra 1, 2, 3 and 4 respectively).

FTIR

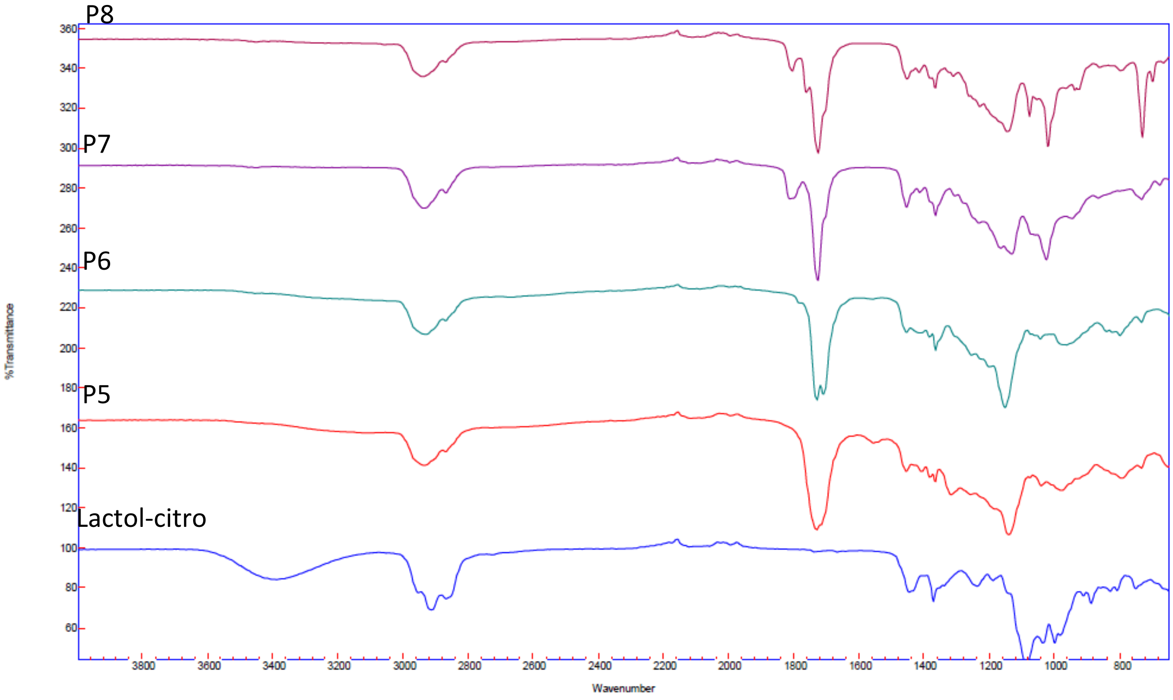

Figure S15. Typical FTIR spectra of P5-P8.

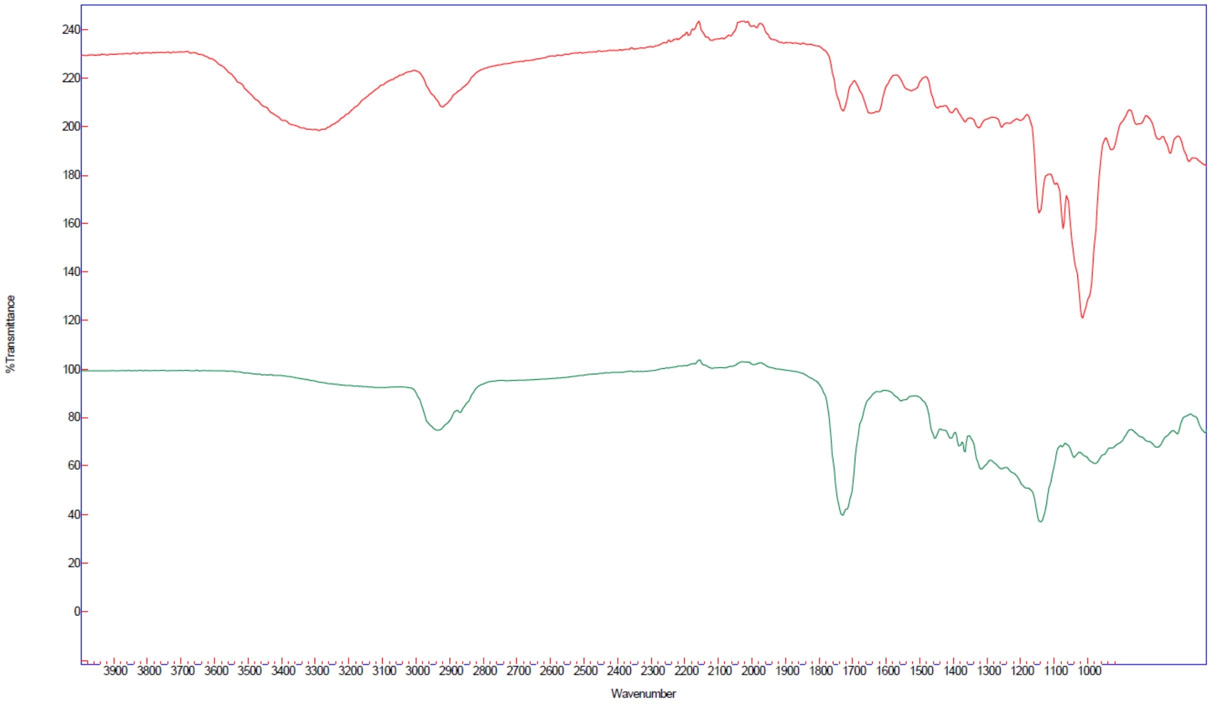

Figure S16. Typical FTIR spectra of P5 before and after enzymatic degradation (green and red, respectively).

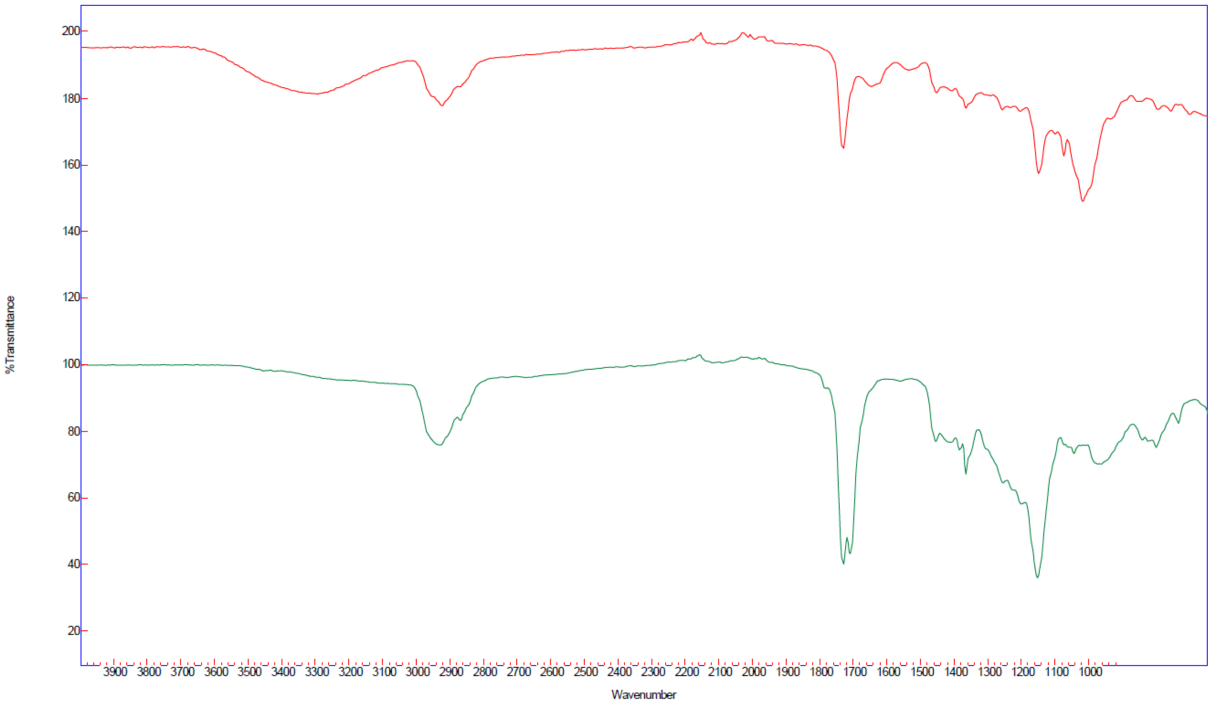

Figure S17. Typical FTIR spectra of P6 before and after enzymatic degradation (green and red, respectively).

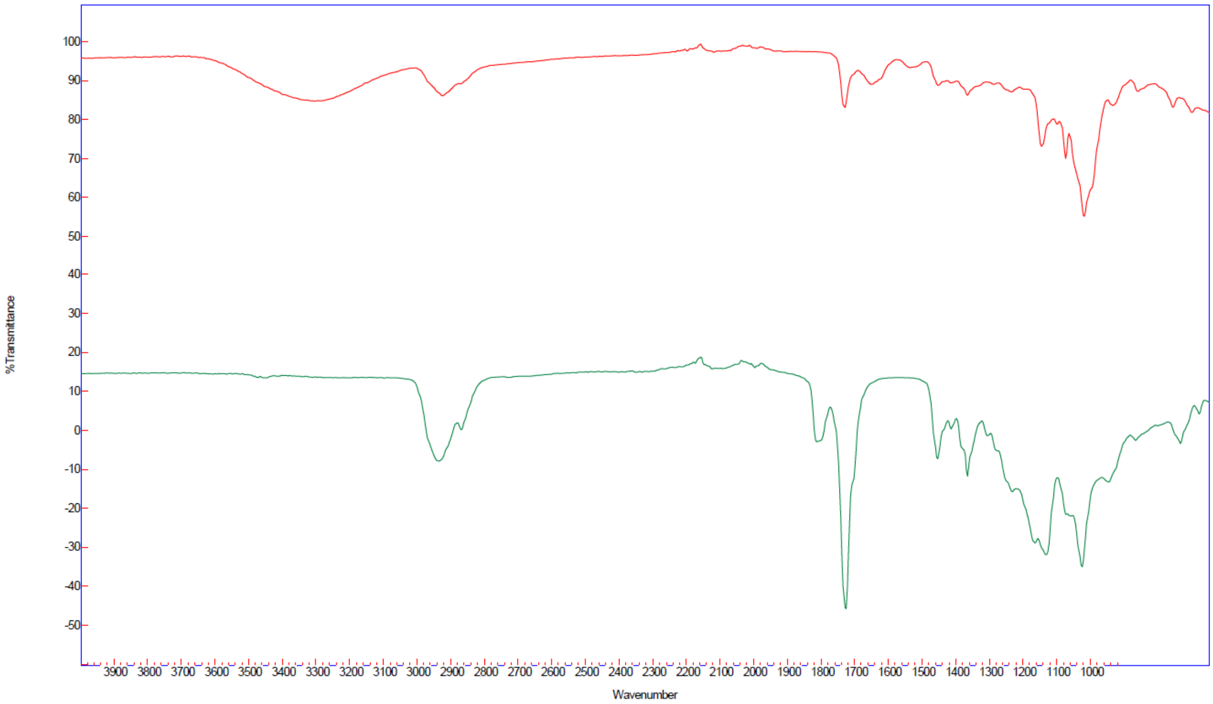

Figure S18. Typical FTIR spectra of P7 before and after enzymatic degradation (green and red, respectively).

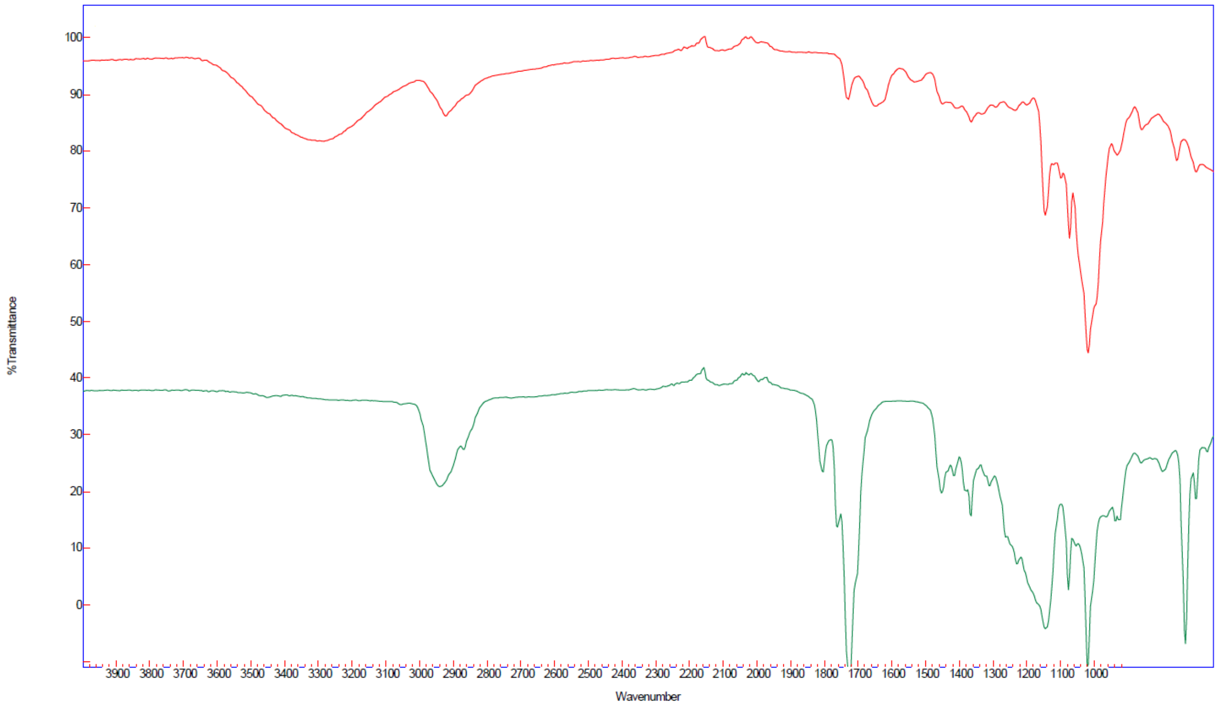

Figure S19. Typical FTIR spectra of P8 before and after enzymatic degradation (green and red, respectively).

DSC

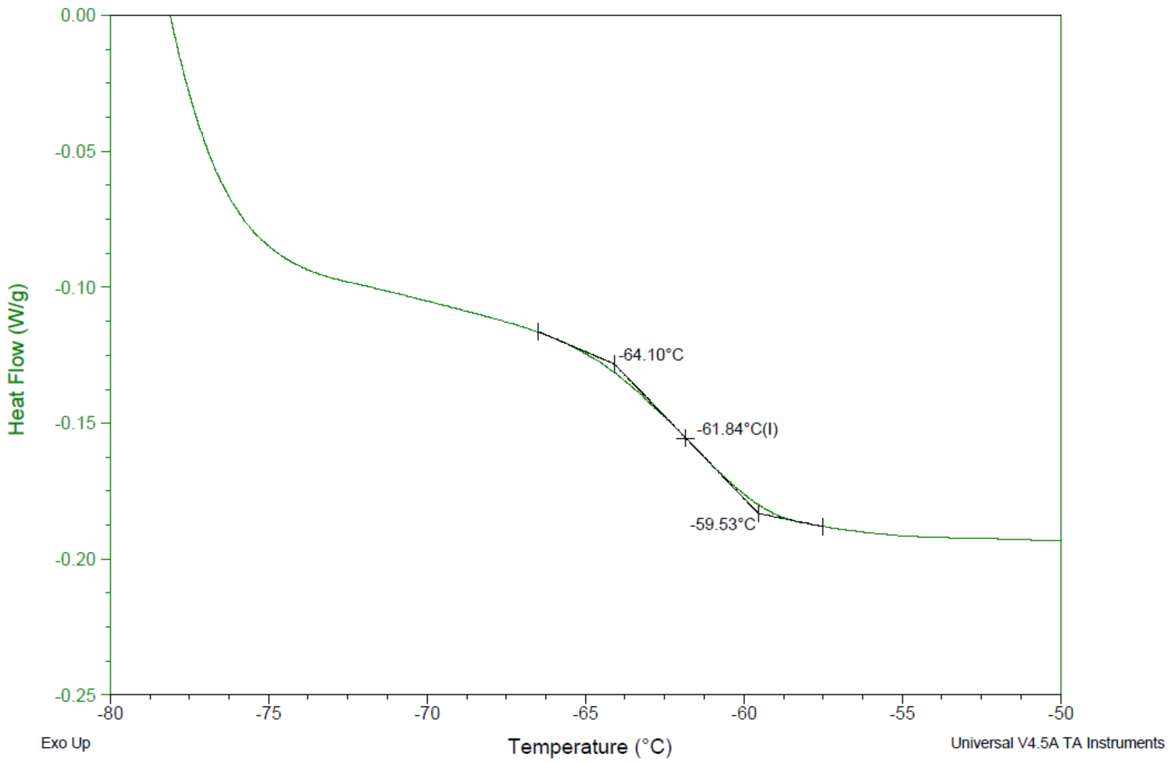

Figure S20. DSC thermogram (3rd heat cycle) of P5, run 1, Table 1.

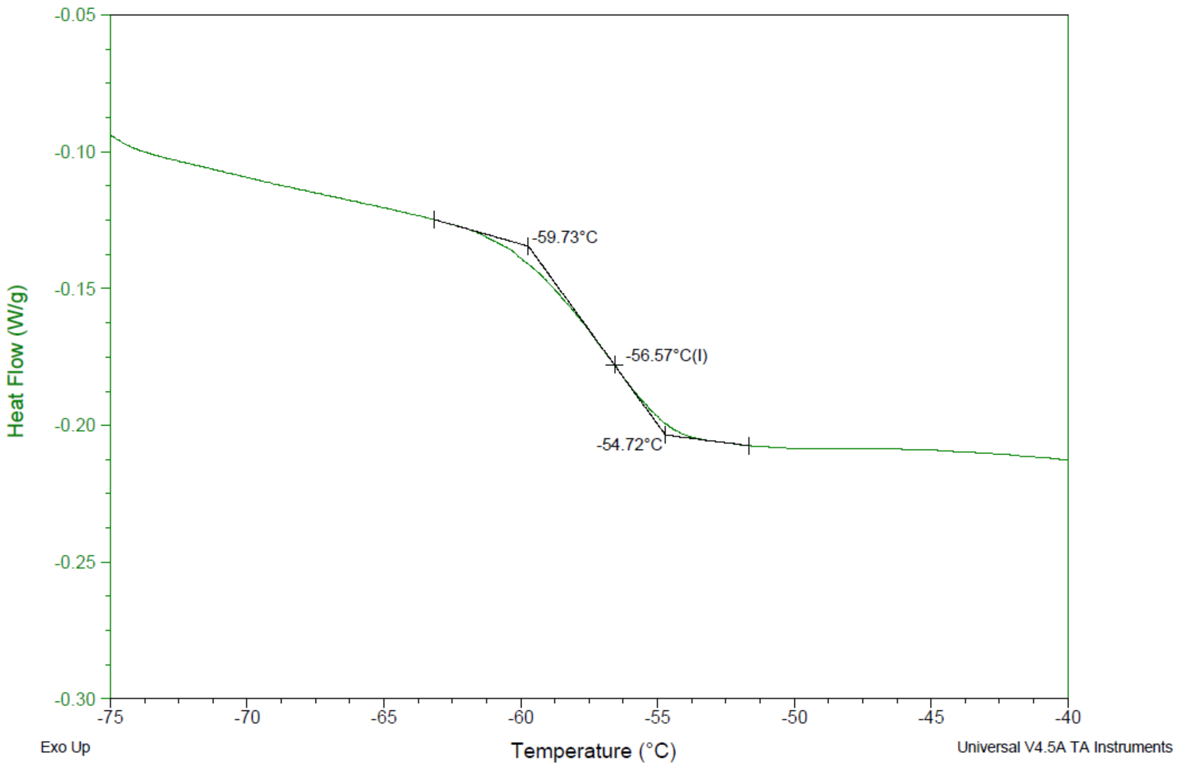

Figure S21. DSC thermogram (3rd heat cycle) of P6, run 2, Table 1.

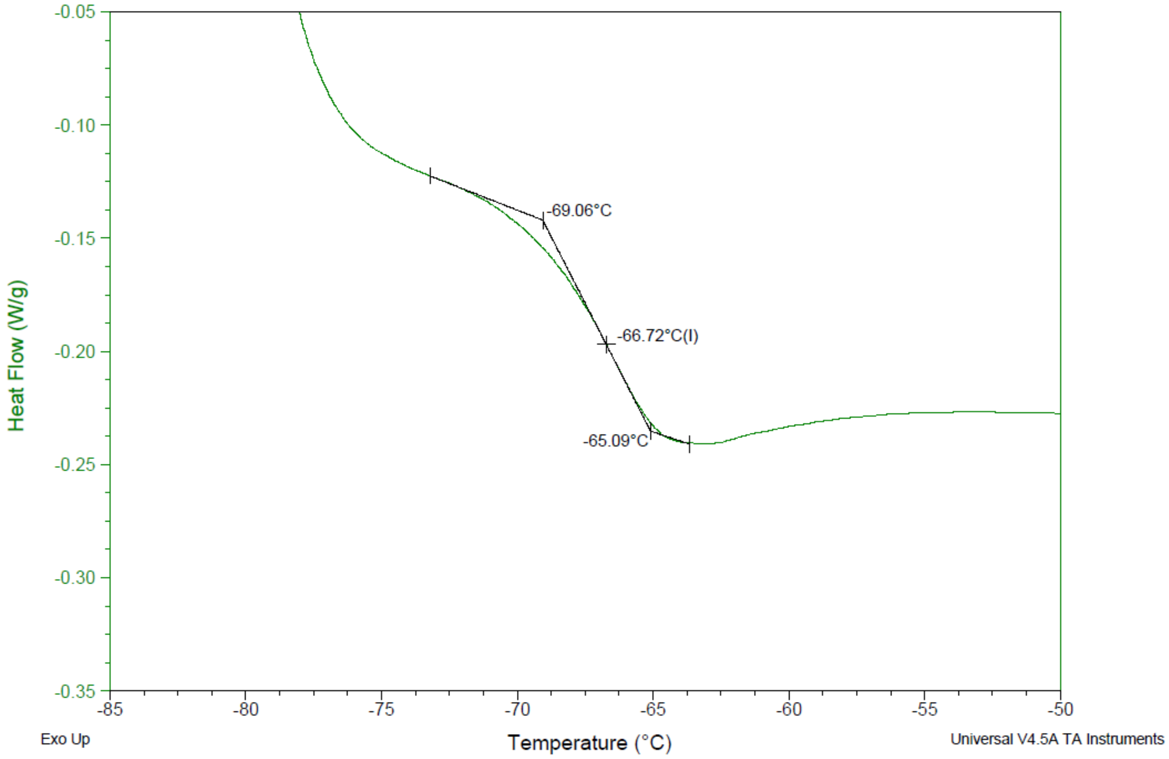

Figure S22. DSC thermogram (3rd heat cycle) of P7, run 3, Table 1.

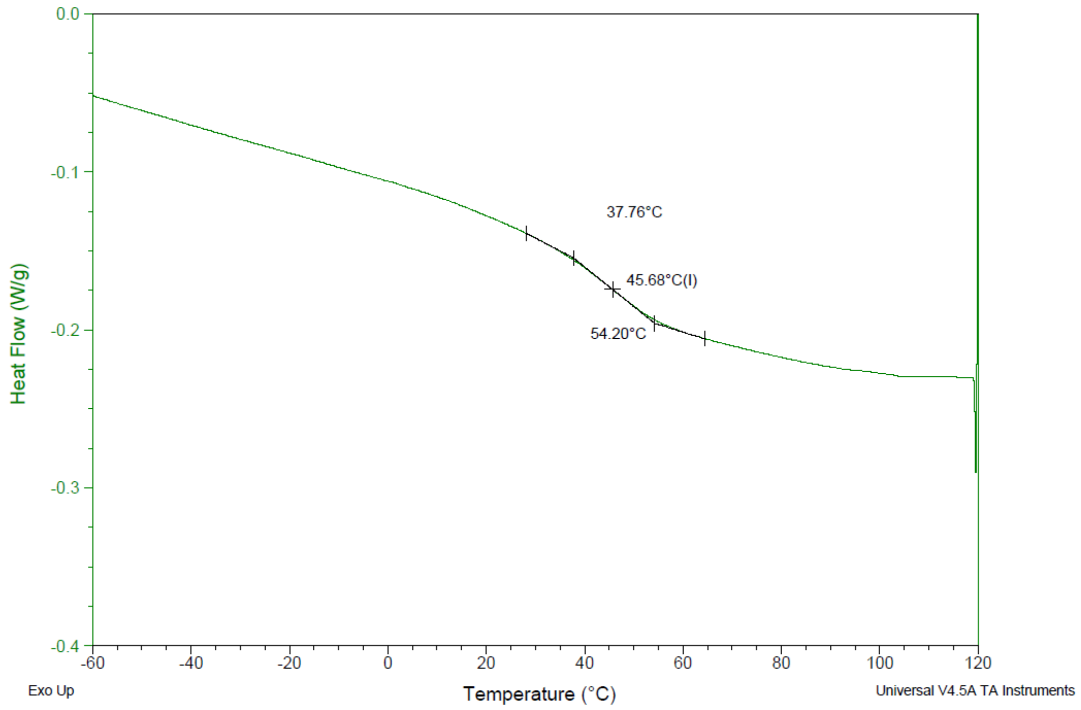

Figure S23. DSC thermogram (3rd heat cycle) of P5 after enzymatic degradation, run 1, Table 2.

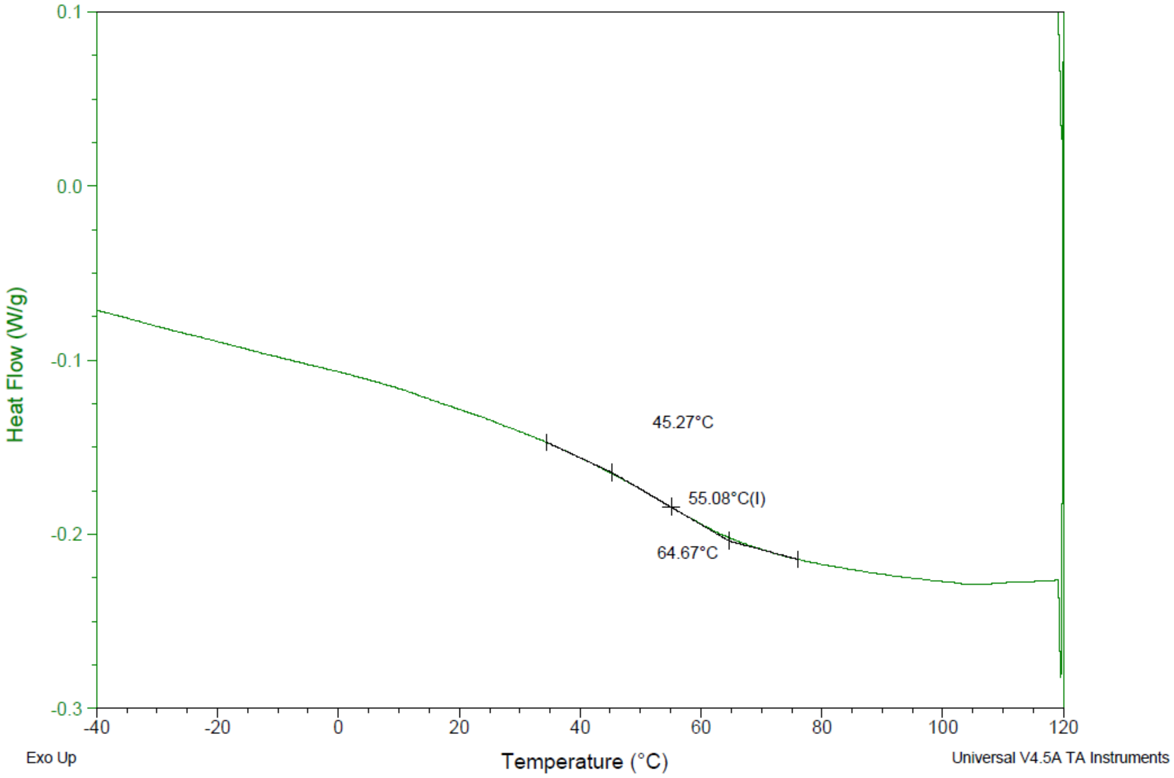

Figure S24. DSC thermogram (3rd heat cycle) of P6 after enzymatic degradation, run 2, Table 2.

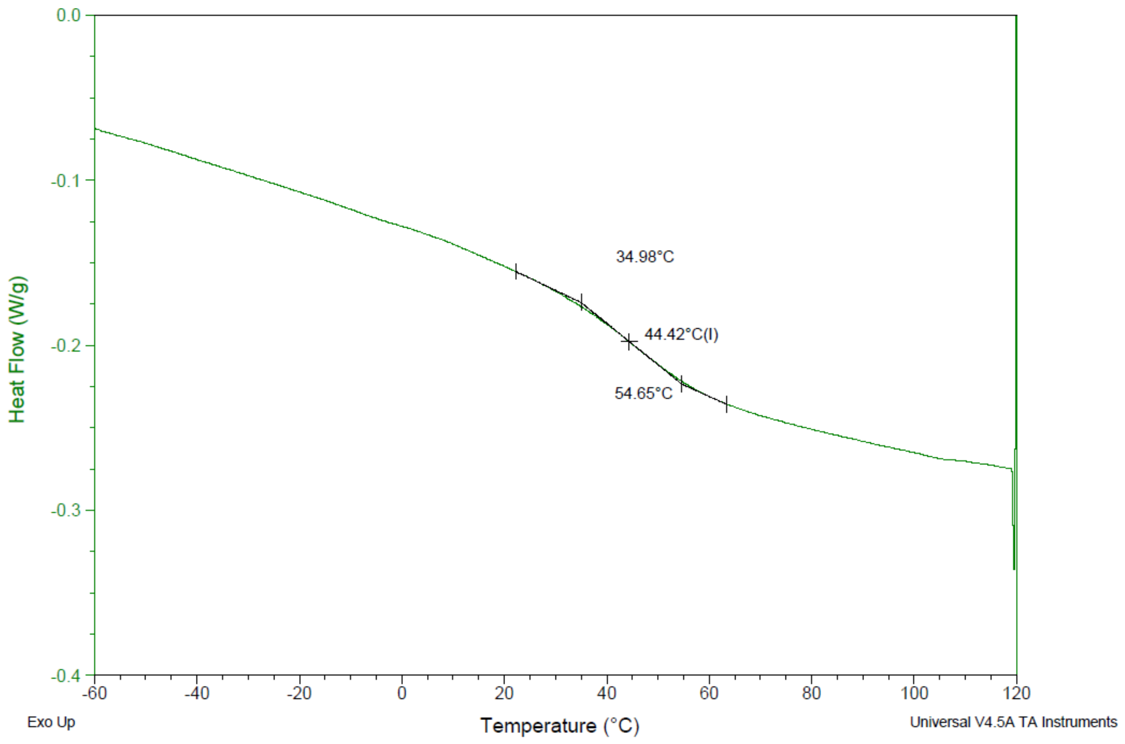

Figure S25. DSC thermogram (3rd heat cycle) of P7 after enzymatic degradation, run 3, Table 2.

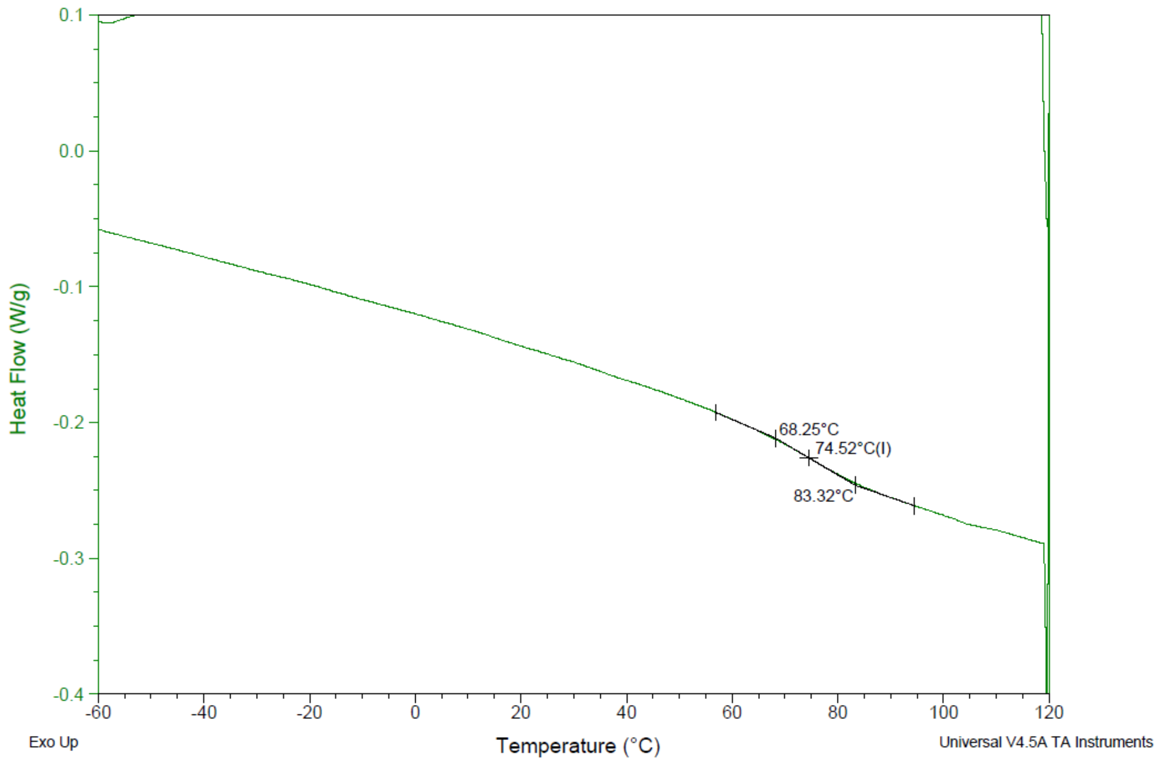

Figure S26. DSC thermogram (3rd heat cycle) of P8 after enzymatic degradation, run 4, Table 2.

TGA

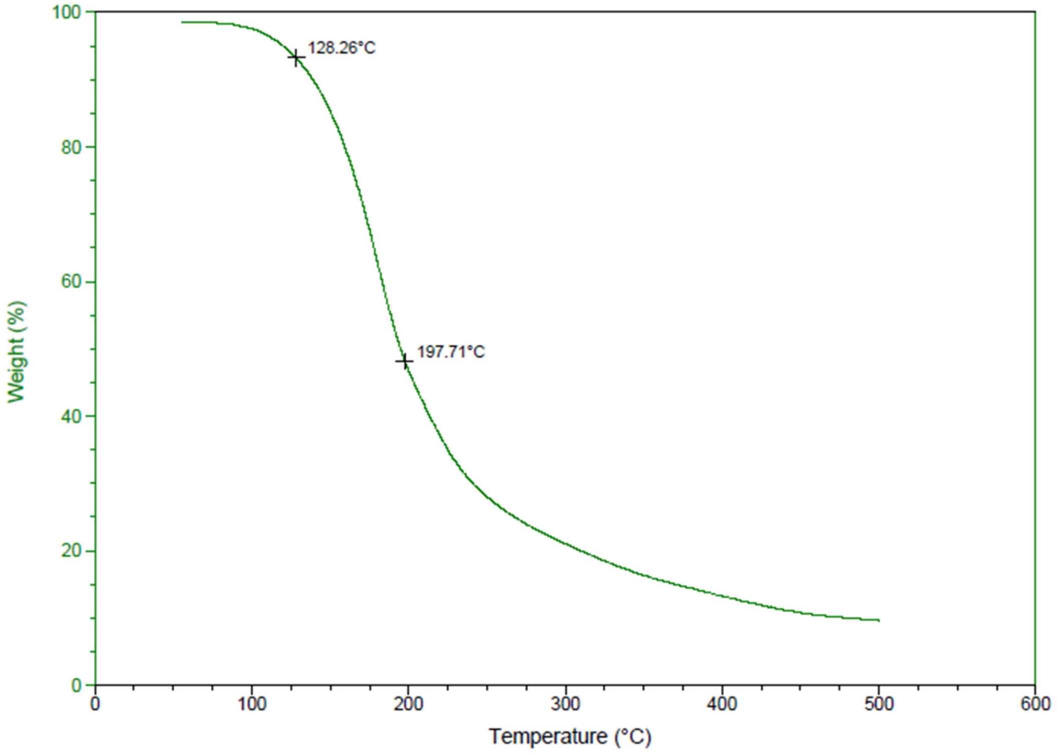

Figure S27. TGA thermogram of P5, run 1, Table 1.

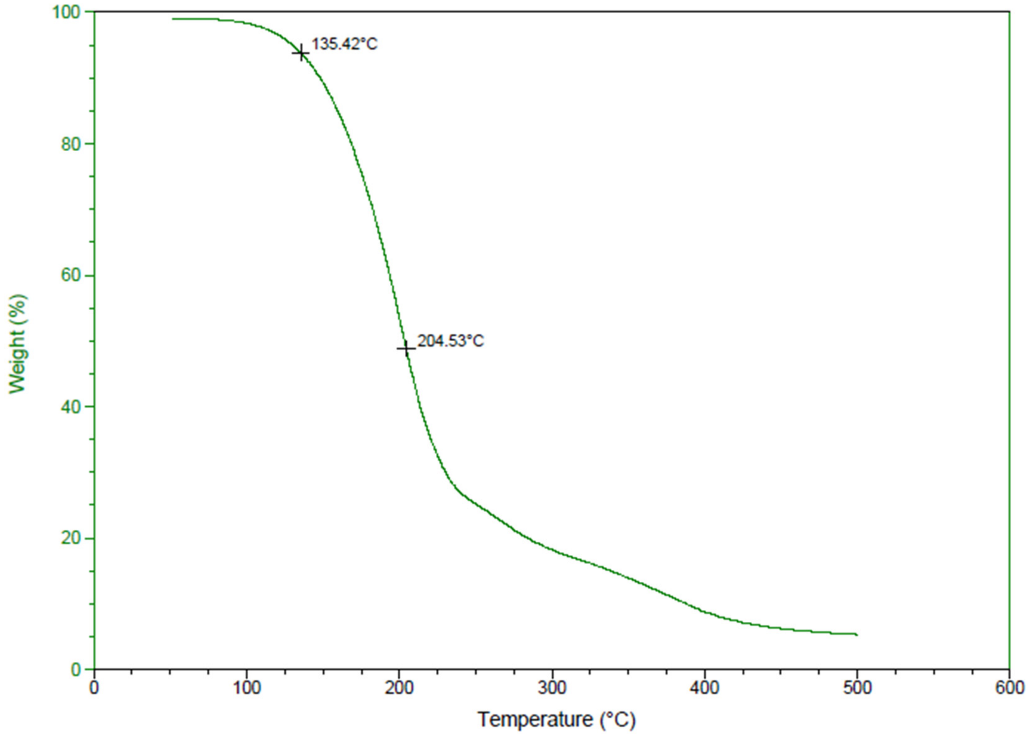

Figure S28. TGA thermogram of P6, run 2, Table 1.

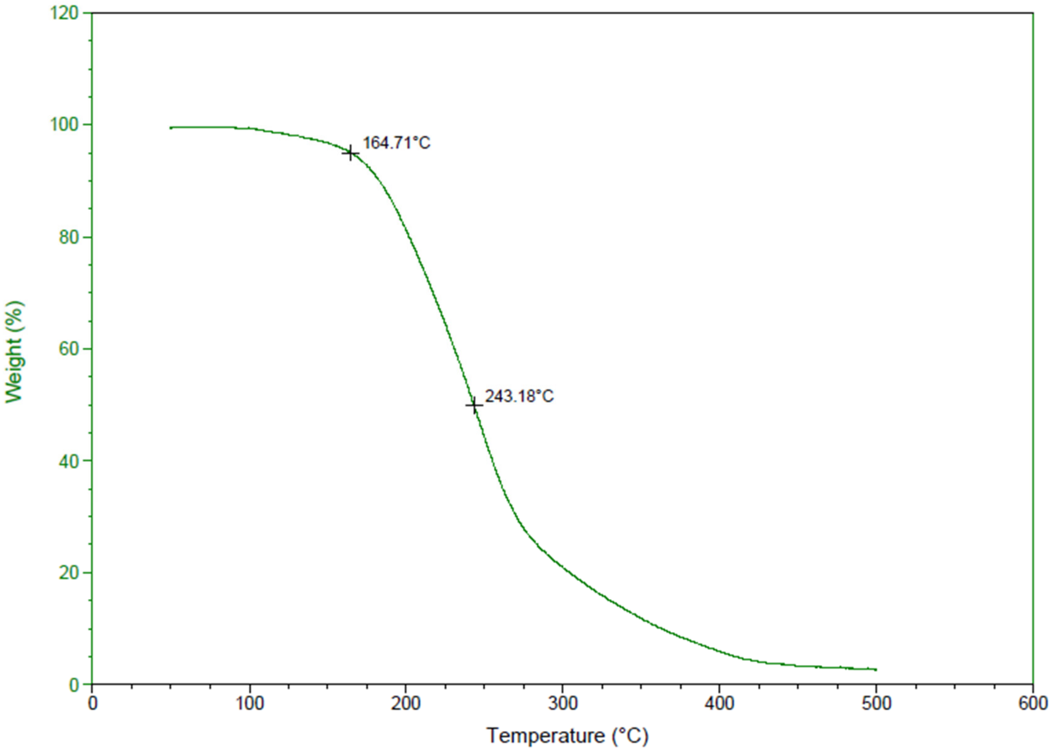

Figure S29. TGA thermogram of P7, run 3, Table 1.

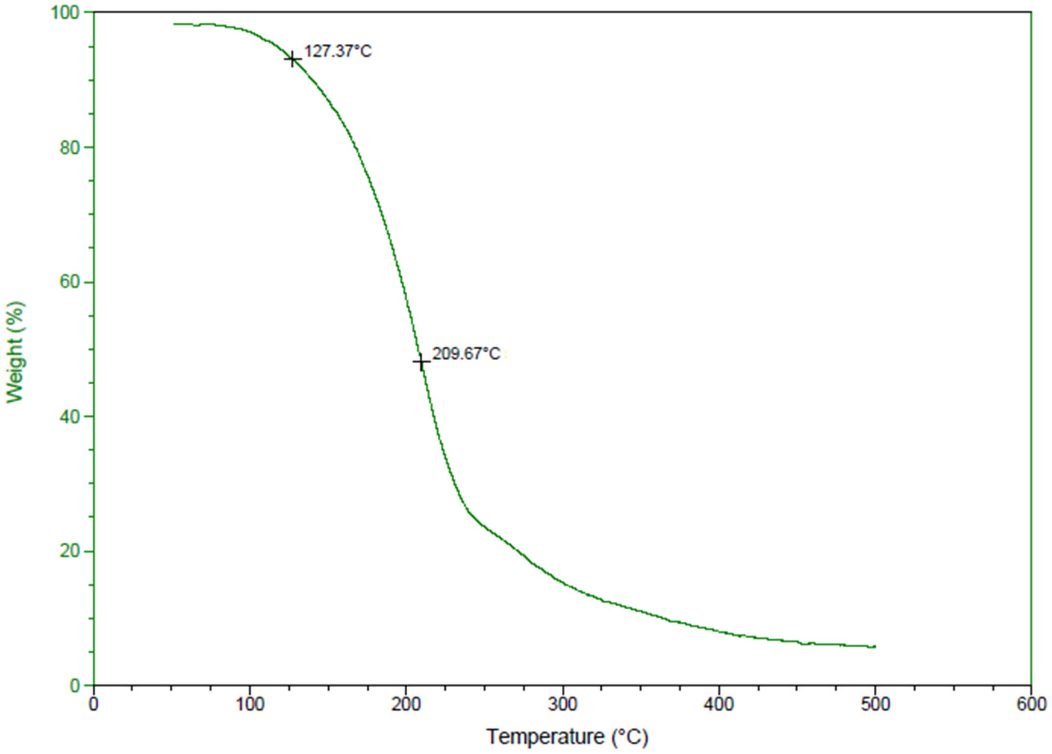

Figure S30. TGA thermogram of P8, run 4, Table 1.

SEC

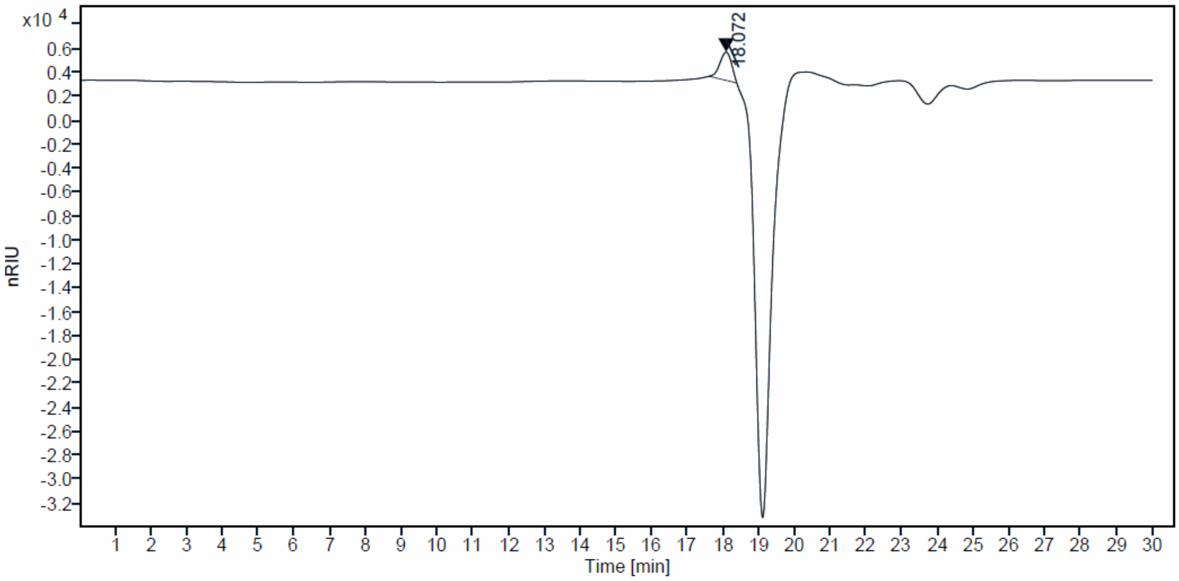

Figure S31. SEC trace of the resulting product of P5 after enzymatic degradation.

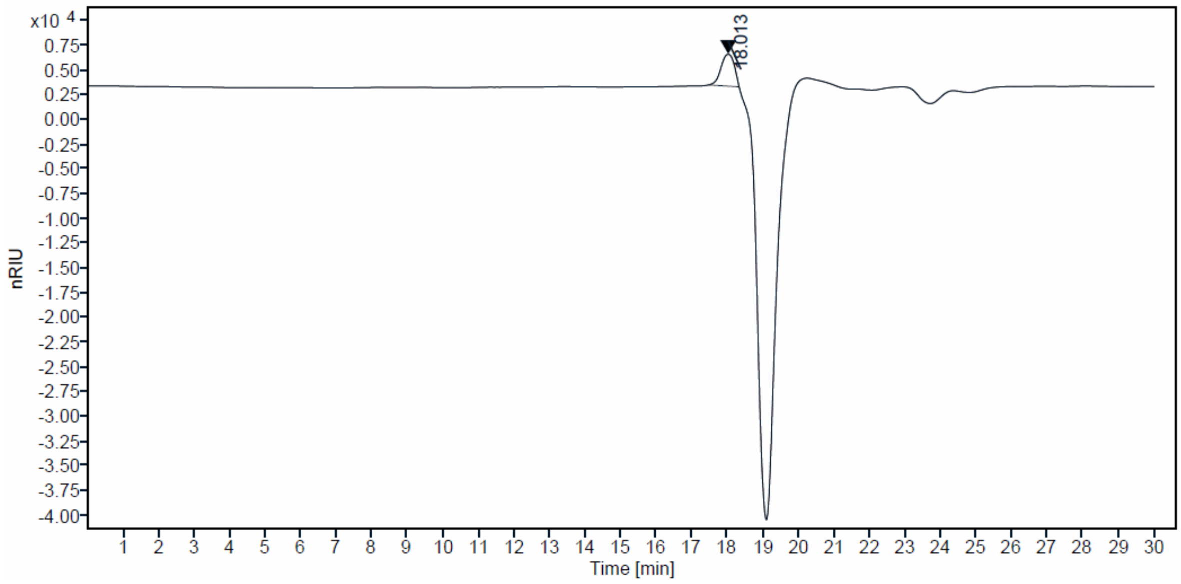

Figure S32. SEC trace of the resulting product of P6 after enzymatic degradation.

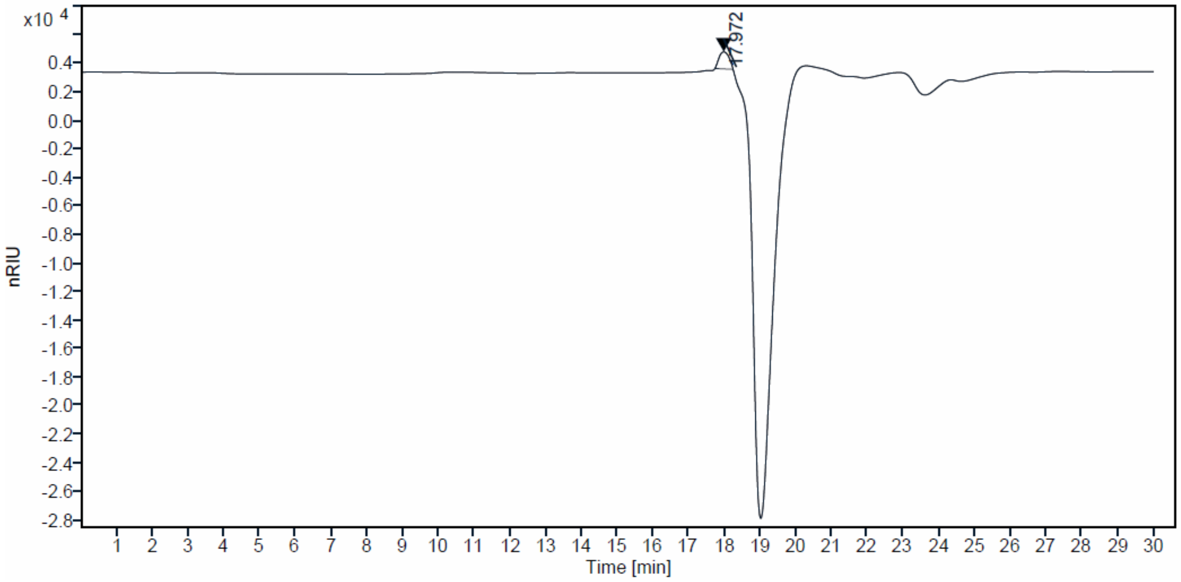

Figure S33. SEC trace of the resulting product of P7 after enzymatic degradation.

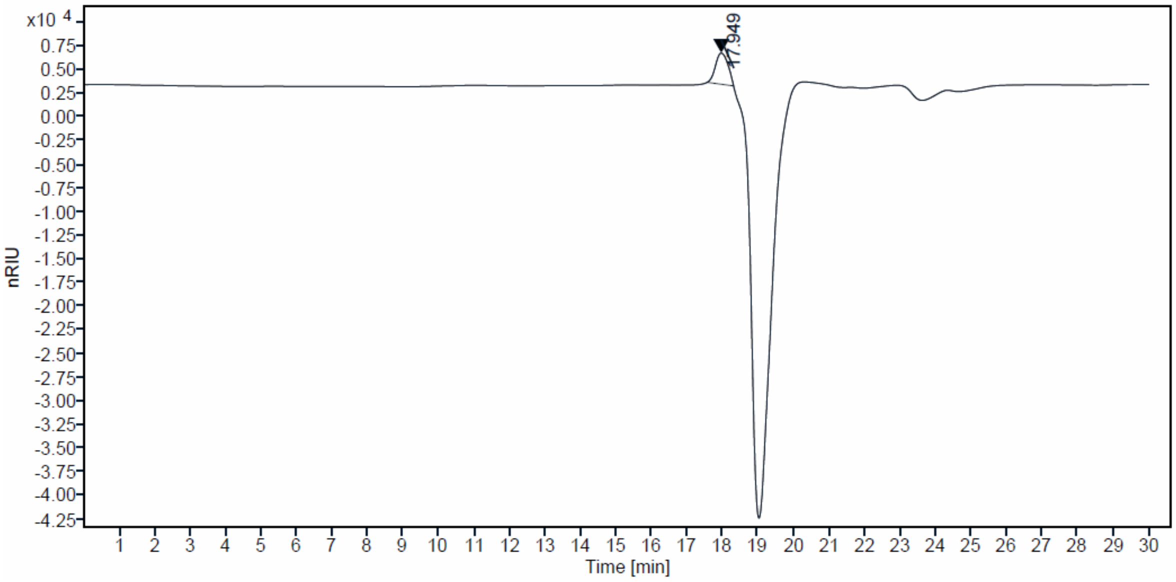

Figure S34. SEC trace of the resulting product of P8 after enzymatic degradation.
